# Intelligent wearable system with accurate detection of abnormal gait and timely cueing for mobility enhancement of people with Parkinson’s disease

**DOI:** 10.1017/wtc.2022.9

**Published:** 2022-06-28

**Authors:** Bao Yang, Ying Li, Fei Wang, Stephanie Auyeung, Manyui Leung, Margaret Mak, Xiaoming Tao

**Affiliations:** 1 School of Civil Engineering and Transportation, South China University of Technology, Guangzhou, China; 2 Institute of Textiles and Clothing, The Hong Kong Polytechnic University, Hong Kong, China; 3 Research Institute for Intelligent Wearable Systems, The Hong Kong Polytechnic University, Hong Kong, China; 4 Shenzhen Research Institute, The Hong Kong Polytechnic University, Shenzhen, China; 5 School of Textile Materials and Engineering, Wuyi University, Jiangmen, China; 6 Department of Rehabilitation Sciences, The Hong Kong Polytechnic University, Hong Kong, China

**Keywords:** freezing of gait, intelligent wearable system, multisensory cueing, real-time detection

## Abstract

Previously reported wearable systems for people with Parkinson’s disease (PD) have been focused on the detection of abnormal gait. They suffered from limited accuracy, large latency, poor durability, comfort, and convenience for daily use. Herewith we report an intelligent wearable system (IWS) that can accurately detect abnormal gait in real-time and provide timely cueing for PD patients. The system features novel sensitive, comfortable and durable plantar pressure sensing insoles with a highly compressed data set, an accurate and fast gait algorithm, and wirelessly controlled timely sensory cueing devices. A total of 29 PD patients participated in the first phase without cueing for developing processes of the algorithm, which achieved an accuracy of over 97% for off-line detection of freezing of gait (FoG). In the second phase with cueing, the evaluation of the whole system was conducted with 16 PD subjects via trial and a questionnaire survey. This system demonstrated an accuracy of 94% for real-time detection of FoG and a mean latency of 0.37 s between the onset of FoG and cueing activation. In questionnaire survey, 88% of the PD participants confirmed that this wearable system could effectively enhance walking, 81% thought that the system was comfortable and convenient, and 70% overcame the FoG. Therefore, the IWS makes it an effective, powerful, and convenient tool for enhancing the mobility of people with PD.

## Introduction

More than 10 million people suffer from Parkinson’s disease (PD) worldwide. A high prevalence of fall incidence from 38 to 68% occurs when a PD patient enters the moderate stage of PD (Koller et al., 1989; Macht et al., 2007; Grabli et al., 2012; Chen et al., 2013). Falls result in huge psychosocial and economic costs to the family and society. The fall incidence is mainly associated with freezing of gait (FoG). FoG is a sudden transient inability to walk, showing Bradykinesia (slowness of movement), hypokinesia (reduced movement excursion), and/or akinesia (difficult to initiate a step or increase variability of continuous steps) (Blin et al., 1991; Lewis et al., 2000; Bloem et al., 2004; Bächlin et al., 2010; Daniel et al., 2019). FoG is the most common gait impairment among PD patients. FoG is likely to appear at the initiation of gait when approaching narrow spaces, turning, and even during walking, especially when performing a concomitant simultaneous activity (Nutt et al., 2011; Virmani et al., 2015).

Physical exercise therapy has been proven in clinical studies to be effective to improve the mobility of PD patients. A 2-week program of physical exercise with acoustic cueing was demonstrated to reduce the freezing score by 22% after the completion of treatment (Fietzek et al., 2014). On the other hand, external sensory cueing, such as acoustic, visual, or tactile cueing, can help PD patients focus on walking (Nieuwboer et al., 2007; Donovan et al., 2011; Fietzek et al., 2014; Martin et al., 2015; Mak et al., 2017; Barthel et al., 2018) and reduce start hesitation (Delval et al., 2014; McCandless et al., 2016). While the effects have been known for many years and clinicians have used these cueing methods for rehabilitation of PD patients, the exact working principle related the “reconnecting” or “retriggering” of the motor cortex of brain that controls of muscle movement remains to be elusive and requires future mechanistic investigations.

Exercise training conducted at clinical tests requires physical attendance of the PD patients, and continuous sensory cueing interferes with the patient’s normal activities. A convenient intelligent wearable system (IWS) equipped with accurate real-time detection of the abnormal gait and timely cueing may solve these two problems. Previous wearable systems can provide real-time gait measurement already and detect the occurrence of FoG. They are equipped with wearable inertia or pressure sensing units (PSUs), including inertia measurement units (Bächlin et al., 2009; Bächlin et al., 2010; Lorenzi et al., 2016), linear accelerometers (Selles et al., 2005), gyroscopes (Catalfamo et al., 2010; Mannini and Sabatini, 2010, 2012; Abaid et al., 2013; Taborri et al., 2015), foot witches (Skelly and Chizeck, 2001; Bae and Tomizuka, 2011; Agostini et al., 2013), and insole pressure sensors (Catalfamo et al., 2008). Among them, the accelerometer is the most common due to its low cost, small size, ease of location, and wireless signal transmission (Lígia et al., 2017). Wearable inertial sensors have been used to characterize episodes of FoG and high sensitivity and specificity in FoG detection were demonstrated. However, the computation of the large quantity of data from accelerometers is complex and time-consuming, resulting in a significant time delay of up to several even dozens of seconds in identifying the occurrence of FoG episodes (Ahlrichs et al., 2016; Kita et al., 2017). To resolve this problem, several researchers tried to predict the occurrence of FoG via changes of electrocardiography and skin conductance (Mazilu et al., 2013, 2015), allowing to initiate a cueing intervention before FoG onset. Yet other walking-related events like sweat and turns disturb the signal and highly reduce the accuracy. In the contrast, PSUs are highly sensitive to reveal transitions of various phases in a gait cycle—single support, swing, double support, heel contact, and push off. Measurements of temporal gait parameters are reliable and accurate (Lopez-Meyer et al., 2011), such as cadence, step time, cycle time, percentage of the gait cycle in swing or single support for each leg, and percentage of the gait cycle in a single limb or double limb support. However, most PSUs have a low fatigue resistance for long-term use and limitation of real-time wireless transmission due to vast amount of generated data.

Therefore, the following key and emerging issues should be resolved before an IWS can be applied in real life. The first is that the sensing and data processing devices used in IWS are bulky, obtrusive, of poor performance, especially, in fatigue resistance. Secondly, FoG is surprisingly difficult to be seen in clinics although it is a common disabling feature in everyday circumstances. Only long-duration FoG episodes are included while short FoG episodes are often ignored, which may lead to inaccurate FoG detection rates (Lígia et al., 2017). Thirdly, the previously reported systems equipped with threshold-based algorithms (Bächlin et al., 2009, 2010; Pepa et al., 2015; Lorenzi et al., 2016) or machine learning algorithms (Mazilu et al., 2012; Kim et al., 2015; Kita et al., 2017; Punin et al., 2017; Mikos et al., 2019) are not ideal for automated detection of FoG, as a long latency may adversely affect the accuracy of real-time detection, and the signal processing operations require substantial computational resources and time. Finally, though different cueing had been developed for PD patients (Ginis et al., 2016; Pieter et al., 2017; Sweeney et al., 2019), the cueing operations should be diversified and meet individual needs. For example, someone may prefer visual cueing, auditory cueing, or both. Moreover, continuous cueing is confined in training sessions as they interfere normal activities. The on-demand cueing can be a better choice in real life.

**Figure 1.**
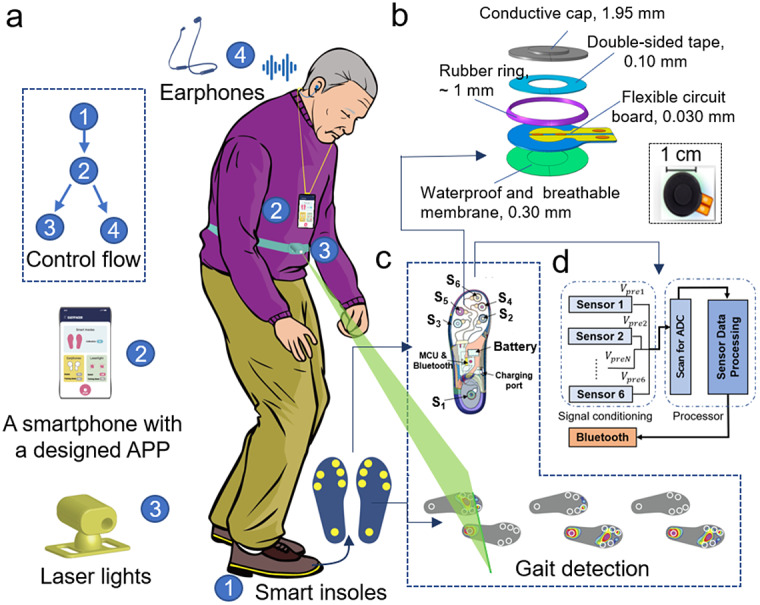
Structure and functions of IWS. (a) Illustration of the IWS worn by an individual with Parkinson’s disease. This IWS consists of a pair of smart insoles that can detect the change of plantar pressure and perform data preprocessing, a smartphone with a customer-made application for data collection, data processing, and providing control signals to cueing devices, and wireless-controlled earphones, laser lights, and haptic vibrators for providing cueing. The systems can detect normal gait and FoG of PD patients in real-time for long-term use, and provide a timely cueing as an external intervention to help the subject overcome FoG. The endurance of smart insoles, wireless earphones, and laser light generators are over 15, 8, and 6 hr, respectively, under continuous working conditions. Thus, they generally can be employed in real-life applications for over 12 h using an automatic cueing mode. (b) Exploded view schematic of a planter pressure-sensing unit. This unit comprises a waterproof and breathable membrane, a FPCB, double-sided tape, a rubber ring, and a conductive rubber cap. The FPCB has two copper electrodes right under the center of the cap concave. When the applied load that acted on the conduction cap reaches the threshold pressure, the conductive cap contacts with the two electrodes due to deformation, the resistance across electrodes decreases from infinity to several Ohms. When the applied load is removed, the conductive cap made from super-elastic rubber carbon can recover quickly and separate from the electrodes, the resistance across electrodes goes back to infinity. Lower-right inset: photograph of the top side of the unit. The sensing unit after fabrication has 2.66 ± 0.16 mm in height, 18.00 ± 0.02 mm in diameter, 23.00 ± 0.05 mm in length, and 0.55 ± 0.15 g in weight. (c) Schematic of the inner structure of a smart insole. The smart insole has a compact design, including a flexible sensing network with six PSUs, a MCU and Bluetooth for data pre-processing and transmission, a lithium battery with a power management module, and a charging port. (d) Block diagram of smart insoles. Smart insoles are responsible for receiving, preprocessing, prestoring sensor data, data transmission as well as network management.

Herewith we design and fabricate a new reliable IWS with accurate real-time automatic detection of FoG occurrence and timely sensory cueing options. The IWS is featured with a novel binary pressure-sensing technology that substantially reduces the amount of data to be transferred and processed. Figure 1a shows the structure and functions of the developed IWS. This system comprises a pair of smart insoles for sensing the pressure pattern of the sole in various gait phases and data preprocessing, a smartphone with a specially designed application for data collection, data processing as well as eliciting control signals to the cueing devices, wireless-controlled auditory cueing via earphones, and visual cueing via laser lights. This IWS can monitor the gait of PD patients continuously and sustainably, detect the onset of FoG automatically and accurately, and provide timely visual and auditory cueing as an intervention when FoG is detected. Clinical trials with two phases were conducted: phase one for collecting data, developing and training the algorithm fort the detection of FoG and phase two for evaluating the performance of the whole system comprehensively.

A total of 29 PD patients participated in 35 trials indicated that the smart insoles can detect the gait well with high accuracy up to 95% and an algorithm developed from the data collect from smart insoles can achieve high accuracy over 97% for off-line detection of FoG. Then, 16 PD patients took part in evaluating the whole system via clinical trials and a questionnaire survey. The clinical trial results show that this IWS can automatically detect FoG occurrence with an accuracy of about 94% and provide sensory curing with a short average delay of 0.37 s from the onset of FoG to the provision of cueing operation. Both video recording and user feedback indicate that the mobility of PD patients has been improved significantly in both walking and overcoming FoG.

## System Design

### Pressure Sensing Units

Each insole has a compact design that integrates a plantar pressure-sensing network with six PSUs. These PSUs are light (≤0.8 g), small (~18 mm in diameter and ~3 mm in thickness), and easy to be embedded in insoles. In addition, a novel cap structure (Figure 1b) was utilized to monitor the change of the plantar pressure with high sensitivity. When the applied pressure is under the designed threshold pressure of the PSU, there is a gap between the conductive cap and the electrodes on the flexible printed circuit board (FPCB), the resistance across these electrodes is large (infinity). When the applied pressure reaches the threshold pressure, the conductive cap deforms and contacts the two electrodes. Meanwhile, the resistance across the electrodes decreases fast from infinity to dozens of Ohms, showing a binary behavior. The threshold pressure can be tuned by adjusting the concave structures inside the cap as well as the conductive rubber materials with different elastic moduli. In addition, wider concave, deeper concave and softer rubbers all can lead to lower pressure threshold. When the applied load is removed, the resistance across electrodes goes back to infinity quickly because the conductive cap is made from super-elastic rubber carbon that can quickly recover. Comparing other pressure sensors, this sensor is unique as it compresses a large amount of pressure–time data into a simple set of binary serial codes 1 and 0 with a predefined pressure threshold value. This feature facilitates a very large compression ratio of the pressure–time series and significantly reduces noise, which greatly improves transmission stability. Moreover, the small PSUs and the management circuit are also easy to integrate into smart insoles.

### Smart Insoles

The locations of PSU were determined according to the foot anatomy and gait features of PD patients. When people walk with normal gaits, there is a contact process for each foot, including the initial strike by the heel, the midstance by the full-foot, and toe-off that is the toe leaves the floor lastly. When PD patients walk with degenerate gaits, the contact sequence becomes different. For example, they are likely to strike the ground using the forefoot firstly when they walk with the torso leaning forward. In humans, normal gait involves single support and swing phases. For the single support phase of the supporting leg, the heel strikes the floor first, the body’s center of pressure proceeding to load fully on the sole in contact with the support surface. The center of pressure moves on the metatarsal heads until the toe leaves the floor. PD patients have a unique foot contact sequence in the single support phase, different from normal people. On turning, they may turn left/right in short steps using the inner/outer border of the forefoot as the supporting region (Supplementary Figure S1). To detect the broad spectrum of gaits, six PSUs (Figure 1c) were utilized and arranged at different contact points according to the plantar-pressure distribution (Shu et al., 2010; Low et al., 2020; Wang et al., 2020) and the above pathological gait characteristics. One PSU (S_1_) was set at the heel and five at the forefoot (S_2_, S_3_ and S_6_ at the metatarsal heads, S_4_ and S_5_ to detect foot-inversion and the foot-eversion). A series of smart insoles over 50 pairs were developed with different sizes and different pressure thresholds of PSUs due to the difference in terms of sizes of feet, weight, and the user habits of PD patients. The sizes of smart insoles range from 36 to 43 in European size. As well as the location of the PSUs will be slightly changed according to the plantar bony protrusion and the pressure distribution. The pressure threshold of S_1_ located at the heel is the highest due to the largest impact effect, the threshold of S_2_ and S_3_ and S_6_ at the metatarsal heads are in the medium, and S_4_ and S_5_ are the lowest. If the shoes are tight, the pressure threshold of PSUs can be adjusted via selecting smart insoles with different pressure thresholds. For our clinical trials, the pressure threshold of S_1_ located at the heel is about 50 kPa and that of S_4_ and S_5_ located at the forefoot is 20 kPa. There are different versions with different pressure thresholds of the other PSUs at the metatarsal heads, such as, one of 35 kPa for the subject with heavyweight or tight shoes, one of 20 kPa for the subject with lightweight, and others in the medium for general cases. Thus, the PD subjects could walk with suitable smart insoles during clinical trials. The smart insoles receive, process, prestore the data detected by the sensors, as well as data transmission and network management (Figure 1d). After signal conditioning of the sensors, the signals are digitized in the embedded A/D converters. The scanned data are then preprocessed in a processor like a microcontroller unit (MCU) and sent to the Bluetooth module for timely transmission to the smartphone installed with a specially designed application (APP).

### Sensory Cueing Devices

Multisensory cueing devices, including auditory and visual cues, were provided to the PD patients when necessary. One cueing option was selected for the patient each time depending upon personal preference. A pair of wireless-controlled commercial earphones, a customer-made laser-light generator were utilized and controlled automatically by an APP that was specially designed for this IWS. Different pieces of music with various rhythms and beats could be chosen in the APP and played via earphones. The rhythm was set individually at 1.1 times the normal walking rhythm of the PD patient (Dalla Bella et al., 2018). The wireless-controlled laser-light generator projects a line in front of the patient and can be fixed on the waist through an elastic belt or fixed on the top of shoes via shoelaces. The project angle and distance of the line on the ground can be adjusted according to individual size and preference. Moreover, the wireless-controlled laser light generators can be fixed on top of the shoes or at the waist level.

### Customer-Made APP

An Android APP has been developed to manage the smart insoles, collect and store data from smart insoles, process data, monitor gait, detect FoG, and wireless-controlled earphones and laser light generators. These wireless-cueing devices can be controlled based on two modes of continuous cueing mode and automatic cueing mode. The continuous cueing mode provides continuous cueing regardless of the gait signals, which can be utilized to help PD patients familiar with the cueing. The automatic cueing mode is utilized to control the cueing devices upon signals received from the smart insoles, which can provide on-demand cueing. Once the onset of FoG was determined automatically according to the developed algorithm, the controlled signal will be sent to trigger the cueing devices. Moreover, in this APP, various rhythmic tones with beats (i.e., 80 bpm) can be selected by users and played on the earphones.

## Characterization

**Figure 2.**
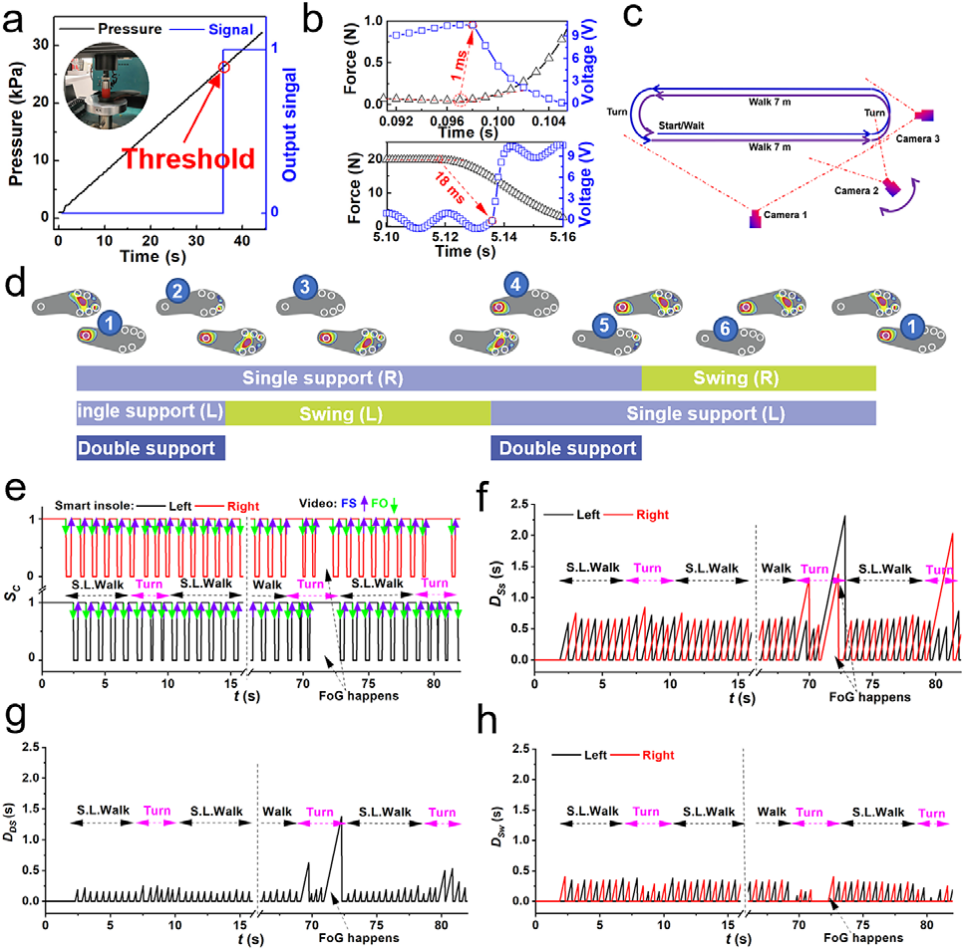
Performance characterization of the IWS. (a) Illustration of the pressure threshold of the PSU embedded in smart insoles. When the applied pressure reaches the threshold, the output of the PSU changes from 0 to 1. Here, the compression of the PSU was performed on a material testing machine (Series 5567, Instron, America) with a loading speed of 0.5 N/s (~0.7 kPa/s). In addition, the diameter of the loading head is ~3 cm. (b) Illustration of the response time of PSUs under cyclic compression. These tests were performed on a material machine (Series 5944, Instron, America) installed with a load cell and a coupled voltage divider circuit (Supplementary Figure S2). The applied force and the resistance change of the bare PSUs were recorded simultaneously. The loading and unloading speeds are the same as 35 mm/s. In addition, a sampling rate of 1 kHz was chosen so that it is enough high to capture the sharp changes of the applied force and the divided voltage across the bare PSU. (c) Schematic of the walking experimental setup for collecting the data from this IWS and videos taken from three cameras. The walking track includes four straight-line walks (S.L. Walk) and four turns. Three cameras are utilized for recording the gait. (d) Schematic of six phases of normal gait including heel strike of the right foot, toe-off of the left foot, mid-standing of the right foot, heel strike of the left foot, toe-off of the right foot, and mid-standing of the left foot and key parameters of single support, swing and double support. (e) Comparison between gait phases detected by IWS and professional observers, showing that the FS and FO detected by IWS have an excellent agreement with those of video observations. Here, the subject walked without and with FoG, and the corresponding results are shown in the left and right half curves, respectively. The subject had 62 kg in weight, a height of 165 cm, an age of 70 years old, a shoe size of 42.5, and an H & Y stage of 1.5. (f–h) The corresponding duration of single support, double support, and swing, respectively.

The PSU has binary outputs (Figure 2a) via a management circuit embedded on smart insoles. When the applied pressure on the PSU reaches or exceeds the designed pressure threshold, the output is 1. Otherwise, the output is 0. These fabricated PSUs can work over 1,000,000 compression cycles under a peak pressure of 25 kPa at a loading frequency of 3 Hz, and even 600,000 compression cycles with a peak pressure at 200 kPa. The excellent durability and fatigue resistance ensure a wearing life over 4 months based on daily walking ~8,000 steps per day. The electromechanical response of the bare PSUs is illustrated in Figure 2b and Supplementary Figure S2. Supplementary Table S1 summarizes the results of the bare PSU measured in the tests. The response time of bare PSUs ranges from 1 to 2 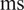 in the loading process and 17–23 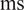 in the unloading process. To evaluate the accuracy of gait detection, PD patients wore the IWS and performed tests with four times straight-line walks and four turns (Figure 2c). Their gait phases will be recorded by the IWS and three cameras simultaneously. There are six phases of normal gaits (Figure 2d), which can be detected by using the distribution of the plantar pressure and visual observation. For each foot, the reaction force between the foot and the floor is cleared, meaning the foot is in the swing phase. Reversely, the foot is in the single support phase. When both feet are in contact with the floor, double support happens. Therefore, there are two key moments, including foot-strike (FS) and foot-off (FO), meaning the initial and end contact between the foot and the floor, respectively. These key moments are independent of the walking styles and can provide enough information for temporal analysis. Afterward, two independent observers analyzed the recorded videos and extracted the timeline of the events of FS and FO. When people walk with normal gaits, the PSU under the heel will be activated in heel contact of the leg’s single support phase. The raw signals detected by six PSUs of the left and right insole are shown in Supplementary Figure S3A,B, respectively. When PD patients walked with abnormal gaits, any PSU embedded in smart insoles may be activated first. Thereby, six PSUs in the insole, forming a sensing network, can detect the pressure distribution easily via the statue of connection and disconnection of each PSU. At any instant of the gait cycle, all output signals are analyzed to detect the key events of 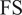 and 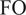 . The combined signal 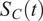 can be given by
(1)

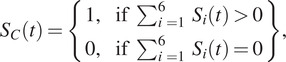

where 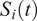 represents the binary output signal of the 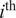 PSU. When 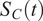 changes from “0” to “1,” 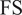 occurs, and when 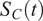 changes from “1” to “0,” 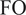 occurs. For each foot, when 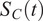 is “1” means the reaction force is above the threshold value of the PSUs so that the foot is in the state of single support, and reversely, when 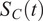 is “0,” the foot is in the state of swing. The combined signals from PSUs were found to be well consistent with those extracted from video shots (Supplementary Figure S3C) when the data extracted from video shots were also sampled at the same frequency of 32 Hz to the IWS, showing high accuracy of up to ~95% compared to the video observation. Similarly, the IWS also can detect the events of FS and FO accurately comparable to the video shots no matter the PD subject walked with or without FoG (Figure 2e). These results indicate that smart insoles are highly sensitive to detecting the gait phases.

Double support is the state when both 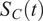 are 1, meaning that both feet are in the state of single support. Once the events of 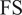 and 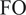 are detected, the peak duration of the 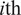 single support ( 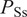 ), swing ( 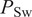 ), and double support ( 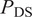 ) can be given by the Supplementary equations S1, S2, and S3, respectively. In addition, the real-time duration of single support, swing and double support can be calculated based on the data from smart insoles, given by the following equations:
(2)

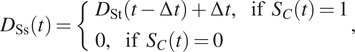



(3)

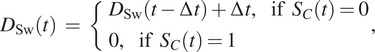



(4)

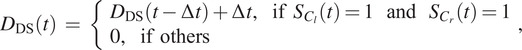

where 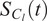 and 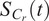 represent the combined signal of six PSUs in the left and right insole, respectively. 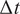 represents the time interval between 2 consecutive data. At 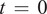 , 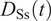 , 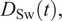 and 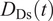 are equal to 0, respectively. When the subject walked with normal gaits, the peak values of 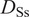 at the straight-line walk are close to those at turns (Figure 2f). While if the subject walked with FoG or other abnormal gaits, the peak values of 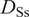 at turns become much higher than those at the straight-line walk. For example, when FoG happens, the peak values can be up to seven times compared to those of normal gait. Correspondingly, the peak values of 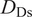 is much higher (over five times) than those at the straight-line walk (Figure 2g). When FoG happens, people with PD may also present with small shuffling steps. Other than long double support, the peak values of 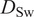 for both feet may be asymmetric at double support. For example, during normal turns, the pivot foot has a long stance and the other foot has small steps for turning. Thus, the peaks of 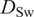 become small (Figure 2h). During FoG, people with PD may walk with small shuffling steps (Supplementary Figure S4). Small shuffling steps induce high values of 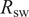 that is the reciprocal of the peak value of 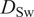 . If these small steps could not clear the foot from contacting the floor, super-long double support will happen. These results are well consistent with the consensus definition of “brief episodic absence or marked reduction of forwarding progression of the feet despite the intention to walk” adopted in the 2010 workshop by clinicians and scientists interested in FoG (Bloem et al., 2004; Giladi and Nieuwboer, 2008).

## Methods

### Methods for FoG Detection

The definition includes FoG manifestations of small shuffling steps, leg trembling, and complete akinesia. Leg trembling but no effective forward motion and complete akinesia implies that both feet cannot be off from contact with the ground so that the reaction force could not be eliminated effectively—the reason for long double support. When the subject walks with small shuffling steps, the stride length becomes much shorter, ranging from millimeters to a couple of centimeters, compared to that of normal gait. The time duration of the swing is reduced. With this understanding, a simple algorithm was constructed to determine FoG.
(5)

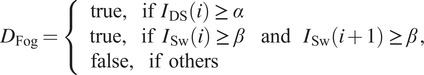

where 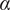 and 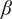 are empirical values. 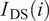 and 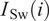 are the indexes of FoG developed from the double supports and the continuous short swing, respectively. If the 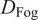 is true, it means that FoG happens. Reversely, it is not FoG.

It is known that gait pattern varies amongst people with PD. Their mobility may decrease beyond the on-medicine period and during the off-medicine period. Thus, IWS requests calibration for all users using a trial involving walking straight-line and making turns. During the calibration test, the subject walks without being affected by FoG. Then, the average pressure peaks for double support and swing can be obtained easily. The indexes of FoG are determined by the following equations. The corresponding data flow is shown in Supplementary Figure S5. And then, the indexes of FoG can be given by the following equations.
(6)

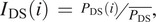



(7)

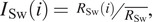

where 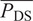 and 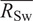 represent the mean value of the peak duration of double supports and the reciprocal of the mean value of the peaks of swings ( 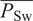 ), calculated by using the data of a calibration test.

**Figure 3.**
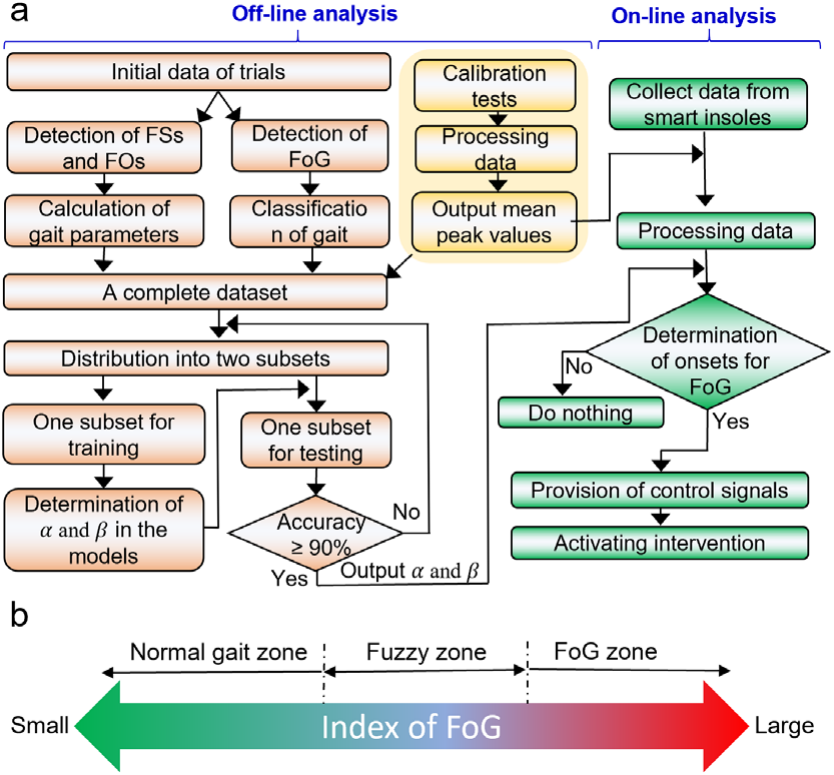
Algorithm for FoG detection and provision of active intervention. (a) Flowchart of the algorithm. The analysis of the initial data of trials (marked in orange) and the calibration test (marked in yellow) is off-line, the detection of FoG and provision of trigger signals are based on an online analysis (marked in green). (b) Schematic of a criterion for determination of the empirical value of 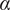 and 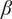 .

In a real-life application, the onset of FoG must be detected promptly and timely for online monitoring to help overcome FoG and avoid falls. The algorithm for detecting the onset of FoG is given below.
(8)

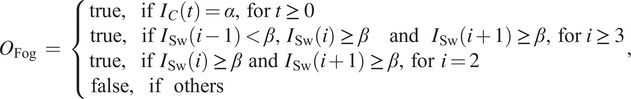

where 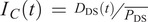 is the index of FoG developed from the double support. And 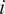 represents the 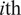 step for each foot. If the 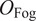 is true, it means that FoG starts. The flowchart of the algorithm for the detection of FoG onsets and intervention is illustrated in Figure 3a. The FoG index as 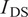 and 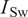 can be divided into three zones: normal gait zone, fuzzy zone, and FoG zone (Figure 3b). The boundaries of the fuzzy zone can be given as the minimum peak value as the low boundary when FoG happens and the maximum peak value as the high boundary for normal gait. The sensitivity, specificity, and accuracy are defined in the Supplementary Information to quantify the performance of the proposed model in detecting FoG, differentiate it from non-FoG, and the degree of veracity, respectively. If both sensitivity and specificity have a high value, the accuracy will also achieve a high value. Thus, a criterion, that is the max sum of sensitivity and specificity is achieved in the fuzzy zone, was utilized to determine the empirical value of 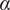 and 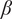 .

Once the onset of FoG is detected, a control signal will be sent to the cueing device. Then the cueing device will be activated so that the subject receives timely intervention to help him/her recover to the normal gait. The cueing device will keep on activating until the IWS confirms the subject’s usual gait has been adopted for 30 s in a continuum.

### Selection of Subjects

Volunteers with PD were recruited from the Hong Kong Parkinson’s Disease Association. The participants were diagnosed with idiopathic PD defined by UK Brain Bank criteria (Daniel Lees, 1993). Their disease severity ranged from stages 1.5–5 of the modified Hoehn and Yahr scale (H&Y), corresponding to mild to severe disease. They were currently on antiparkinsonian medications, were able to ambulate independently with or without walking aids, and the presence of bradykinesia or freezing halted continuous ambulation on both feet on the ground. Subjects were excluded if they had comorbid conditions including musculoskeletal, cardiovascular, respiratory, or neurological disease, uncorrected visual impairment, movement disorders other than bradykinesia or freezing, or cognitive impairment (Montreal Cognitive Assessment score ≤ 24). Subjects participated in the trials included 43 people with PD (31 males, 12 females; 65.3 ± 8.1 years old; 59.7 ± 10.3 kg in weight; 164.4 ± 7.7 cm in height) and one health participant (male; 31 years old; 60 kg in weight; 178 cm in height) who could mimic the pathological gait pattern of PD. The characteristics of the subjects are summarized in Supplementary Table S2. Among them, there are two subjects who walked with FoG frequently in clinical tests and took part twice for training the algorithm and evaluating IWSs, respectively.

### Trial Protocol

Trials were conducted in the laboratory of the university. There are 29 PD patients and one healthy subject participated in clinic trials as the phase one. In this phase, the subject walked with smart insoles and the PSU data and walking video were collected. And then, the algorithm of FoG detection was developed based on the analysis of gait. Another 16 PD patients took part in clinic trials as the phase two. The subject walked with the whole system including smart insoles and wireless cueing devices. The accuracy of FoG detection, the response time, enhancement of mobility, comfortability and other performance of the whole system was evaluated via these trails and questionnaire survey. Subjects attended the trial when they were near the end of the on-medicine state so that the trial spanned through the on-medicine to the off-medicine state for the different walking behavior to be examined. Each trial session lasted around 2 hr and involved a series of tests on walking.

For the walking test, subjects were required to walk twice on a straight 7 m path, which had a horizontal line marked at the start (start-line) and one at the far end of the path (end-line). Three cameras were set up to video-record the gait and posture of subjects—one set at the far end of the straight path, and two laterals to the path.

1. At the beginning of the session, each subject was introduced the IWS and explained the trial procedure. While in sitting, they set the IWS by selecting and connecting devices via Bluetooth with the help of a project assistant.
2. Then the subject stood at the start-line, and started walking at their usual pace upon hearing the instruction of the project assistant, turned after walking pass the end-line, keep walking to the start-line again, turned and repeated the second to and fro along the path. The walking test stopped when the subjects completed the final turn to face the straight path after crossing the start-line. This first attempt provided the data for the IWS algorithm to calibrate the threshold demarcating the presence of FoG.
3. The subjects then resumed sitting on the chair to rest for a few minutes.
4. Subjects then attempted six walking tests in the on-medicine state, and then in the off-medicine state. Afterward, they were interviewed to complete a user-feedback questionnaire, which was listed in Supplementary Section S2. The questionnaire includes three sections: six items on comfort and practicality of the IWS, six items on efficacy, and five items on further improvement for the IWS.

## Results

### Accuracy of FoG Detection

To train and test the algorithm, 29 PD patients and one healthy subject who can mimic FoG participated in clinic trials. During these clinic trials, there are 1,502 FoG with a mean episode of 2.4 s. These episodes of FoG ranged from 0.4 to 31.8 s. The whole dataset consists of gait parameters of 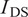 and 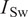 and FoG marker, which was randomly allocated to two subsets for training and verifying the algorithm. In the dataset, the data with/without FoG of each subject were divided randomly and equally. Referring to equations (5) and (8) set for the algorithm, the key parameters of 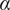 and 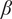 were independent and affected by different FoG appearance. To achieve best accuracy of the detection of FoG, 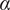 and 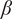 were trained using one subset. And then, the performance of detection in terms of sensitivity, specification and accuracy were verified using the other subset.

The training and testing results show high sensitivity, specificity, and accuracy in FoG detection (overall 97%) (Supplementary Table S3). And the obtained values of 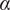 and 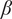 are 4.7 and 4.5, respectively. Meanwhile, the detection of each subject can achieve high accuracy all over 90% when 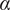 and 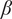 are 4.7 and 4.5. And then, the combination of 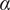 and 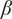 were utilized in the training and testing subset for verification. The results (Supplementary Table S4) also showed high sensitivity, specificity, and accuracy (about 95% or higher). It is noted that the obtained values of 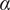 and 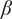 (4.7 and 4.5) were trained and tested based on the data of 35 trials. These values can be utilized as default values for the application. To achieve higher accuracy for individual and follow their user’s habit, the values of 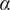 and 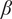 can be adjusted manually in the APP.

Through the above off-line analysis, the optimal values of 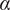 and 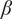 unique for the subject were determined. They were utilized in the online monitoring of the onset of FoG. Afterward, 16 PD subjects conducted 95 walking tests to evaluate the whole system. The accuracy of online FoG detection also was evaluated. After the first attempt, the subject walked with auditory and visual cueing alternately. During the tests, the subject experienced on-medicine and off-medicine states. There were 662 FoG reminders triggered in the IWS, and 623 FoG detected by human inspection of the video shots recorded in the walking trials. There was 94.1% of correct detection, 5.9% of over detection, and no under detection, respectively, confirming high accuracy in FoG detection (Table 1).

**Table 1.** The detection results for various events obtained from video and IWS

	Events (no. occurrence)			
	Total no.	LDDS	SSW_L	SSW_R
Video counts	623	384	130	109
IWS detection	662	412	132	118
Correct detection	623 (94.1%)	384 (93.2%)	130 (98.5%)	109 (92.4%)
Over detection	39 (5.9%)	28 (6.8%)	2 (1.5%)	9 (7.6%)
Under detection	0 (0.0%)	0 (0.0%)	0 (0.0%)	0 (0.0%)

*Note:* LDDS, SSW_L, and SSW_R represent the events of long double support, continuous short swings of left leg, and continuous short swings of right leg, respectively.

### Response Time

The schematic data flow of the IWS determines the response time, from the onset of FoG to activation of cueing, is illustrated in Supplementary Figure S6. This response time includes the time durations of sensing and data pre-processing in smart insoles, data transmission from smart insoles to the smartphone, data collection, data processing, FoG determination and sending control signal via the smartphone, data transmission from the smartphone to the cueing devices, determination and cueing activation in the wireless cueing devices. The PSU response time is less than 2 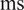 for loading and 20 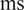 for unloading, thus neglectable in comparison to hundreds of 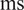 for the gait feature durations of double support, swing, and stance. When the IWS provides visual cueing, the average response time is 0.37 s for long double support and continuous short swings. Yet, there is a relatively large variation in the response time. For example, the maximum and minimum response times are slightly different, such as 0.71 and 0.23 s for long double support (Supplementary Table S5), and 0.73 and 0.21 s for continuous short swings (Supplementary Table S6). Because the wireless transmission via Bluetooth is using data packages, including eight groups of data, for achieving stable transmission. Regarding the sampling rate of 32 Hz, the packaging process can induce over 0.25 s variation. Though there is a variation, the response time of this IWS is the fastest in comparison with other cellphone-based FoG detection systems, such as only the latency of data processing is up to 0.4 ~ 2 s (Mancini et al., 2019) and 0.34 s as a mean value (and a maximum of 0.71 s) in another case (Mazilu et al., 2012).

### Enhancement of Mobility

**Figure 4.**
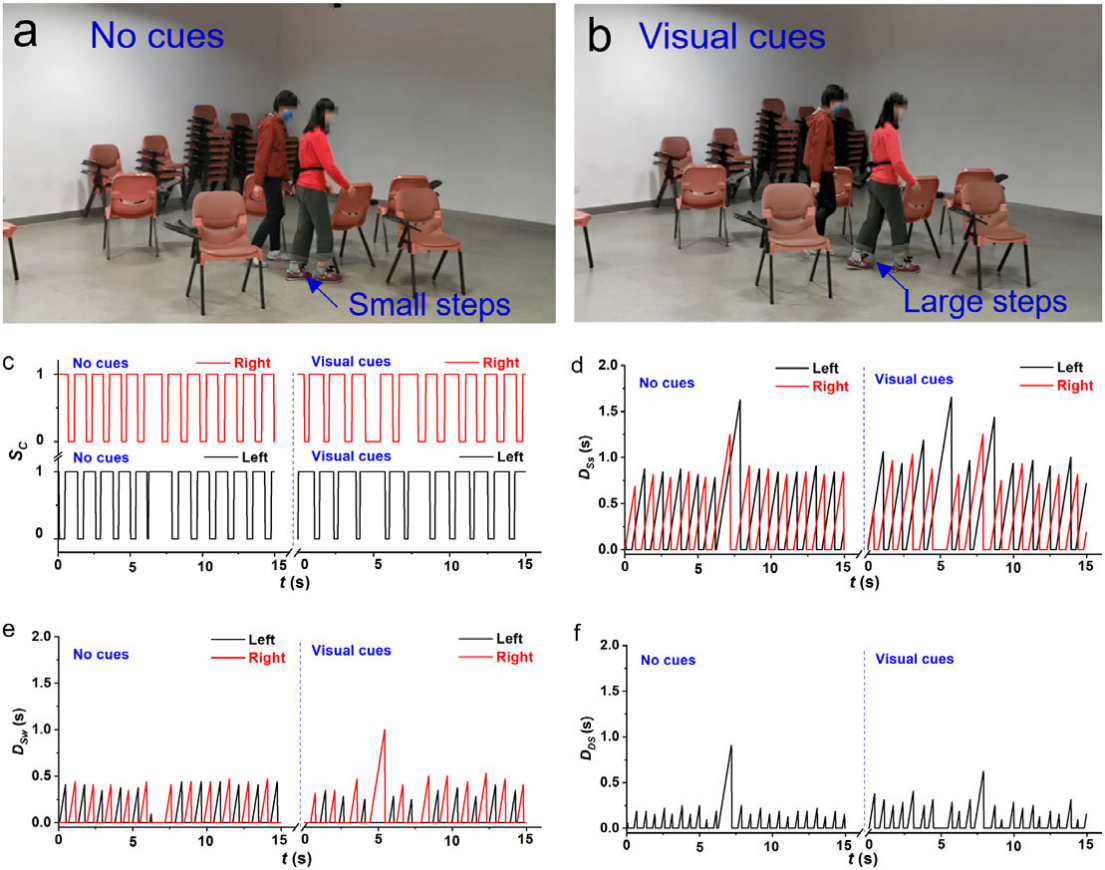
Demonstrations of the mobility enhancement of PD patients in the clinical trial. (a,b) Comparison between walking through a narrow path without and with visual cueing, showing that the subject walked with larger steps when there were visual cueing compared to that when there is no cueing. This observation is from inspection only. The corresponding gait phases and parameters: (c) Combined signal (Sc) of smart insoles, (d) Duration of stance 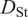 , (e) Duration of swing 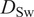 , and (f) Duration of double support 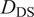 , where the subject with PD had 52 kg in weight, a height of 155 cm, an age of 66 years old, a shoe size of 40.0, and the H & Y stage of 1.5.

To evaluate the efficiency of the IWS, 16 trials that PD patients walked with and without cues were designed and performed. The subjects walking with visual or auditory cueing were video recorded, as shown in Supplementary Movies S1 and S2, respectively. As shown in Figure 4a,b, a female subject walks slowly through a narrow path, blocking by several chairs, with small steps when there were no cueing. And when she walked with visual cueing, she could walk with large steps and go through the narrow path smoothly and quickly (~2.6 s needed), showing that there is an improvement of ~200% in the walking speed. These results indicate that visual curing provided by the IWS can effectively improve the subject’s mobility. Interestingly, this subject’s mobility has improved in the gait length by using visual cueing, but the average walking frequency is the same as that she walks without cueing. Consequently, the calculated single support, swing, and double support show similar results between those with and without visual cueing (Figure 4c–f).

Moreover, the occurrences of FoG are strongly correlated with the subject’s mood and the surrounding environment. Yet, it is difficult to keep the same mood during different tests, which means that it is difficult to present a base line for different individuals. And thus, a questionnaire survey from PD subjects and the gait changes of groups are effective ways to evaluate the wearable system. There are 29 subjects walked without cueing in the phase one and 16 subjects walked with cuing in the phase two. Though during trials, all the subjects walked with smart insoles for data collection, the smart insoles are comfort like common sport insoles (Supplementary Figure S7), featuring in soft and lightweight (~70 g). The good comfortability was confirmed in the questionnaire results (Supplementary Figure S8A), and thus, it is reasonable that the effect of smart insoles on the gait can be neglected. And the gait of PD subjects in the phase one can be treated as a baseline. The direct comparisons between the subjects walked without and with cuing were added. The FoG occurrence of groups who walked without and with cueing indirectly indicated that the FoG occurrences reduced from 44 times/trail (phase one without cueing, 29 trials with 1,502 FoG) to 39 times/trial (phase two with cueing, 16 trials with 623 FoG). There were two PD patients (Subject ID 24 and 28 listed in Supplementary Table S2) who walked with FoG frequently participated in phase one without cueing and phase two with cueing. Comparing to phase one where the subject walked without curing, the FoG occurrence reduced from 6 times/cycle to 3 time/cycle (Subject ID 24) and from 112 times/cycle to 24 times/cycle (Subject ID 28), respectively. These results are consistent with previous results, including that auditory cues which are rhythmic or mimicking footsteps could improve cadence and gait speed (Willems et al., 2006; Young et al., 2016), while visual cues could improve stride length and reduce gait variability(Morris et al., 1996; Lewis et al., 2000; Suteerawattananon et al., 2004; Rocha et al., 2014).

### Individual Gait Variation from Normal Status to FoG

**Figure 5.**
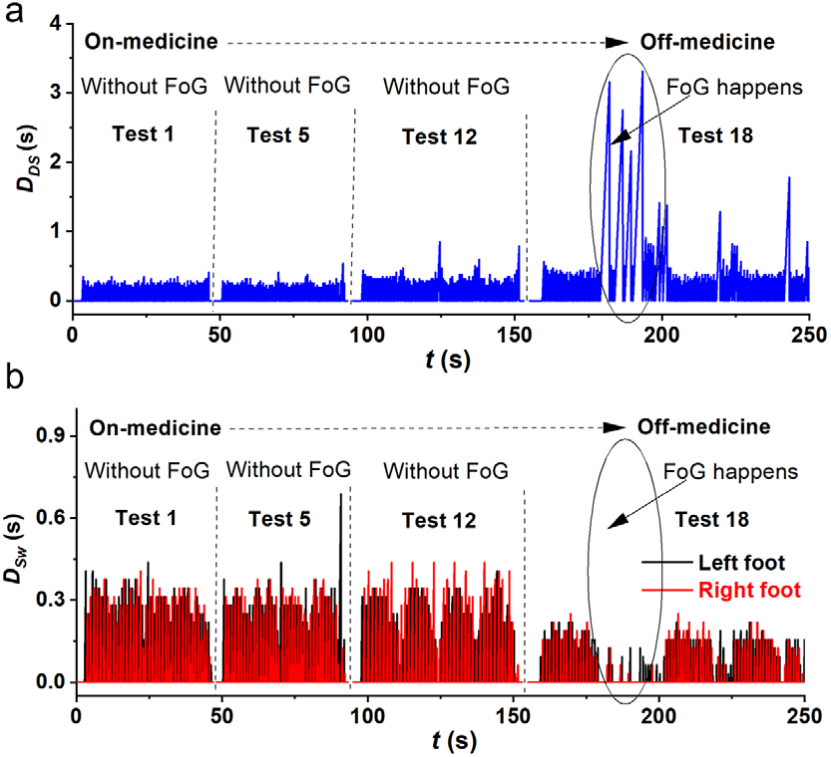
Key gait parameters of an illustrated PD subject, changing from normal status to degenerated status: (a) Duration of double support ( 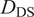 ), (b) the duration of swings ( 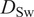 ), where the Subject with PD had 68 kg in weight, the height of 158 cm, age of 73 years old, the shoe size of 41.0, and H & Y stage of 3.0.

Under the influence of medicine, most PD subjects could walk with relatively normal gaits, which are close to or even the same as the gait of healthy subjects. With the gradual decline in efficacy of the medicine, the subject walked with degenerated gaits and even FoG. The gait of a PD subject was monitored from the normal status (on medicine) to a degenerated status when the efficacy of medicine curing has been fully passed (off medicine). The key gait parameters like the durations of double support and swings have obvious changes. For example, the duration of double support increases drastically, up to eight times from on medicine to off medicine (Figure 5a), while the duration of swings decreases (Figure 5b).

### User Satisfaction of IWS

**Figure 6.**
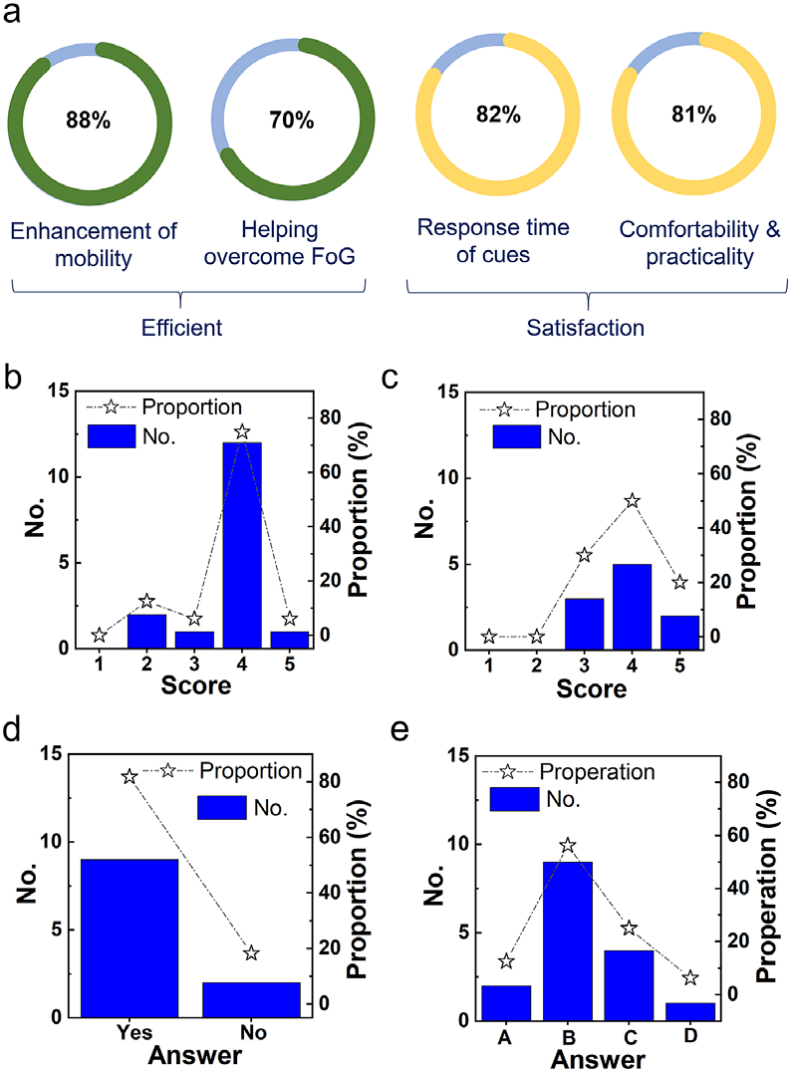
Overview of IWS through questionnaire from 16 participating PD patients. (a) Efficient and user satisfaction of IWS, including enhancement of mobility, helping overcome FoG, satisfaction on the response time of cueing, and comfortability and practicality. The comfortability and practicality includes comfortable of the insoles, the fixing position and the weight of laser light devices, and ease to wear and use the APP. (b) The level of improvement of walking: 1—absolutely no improvement, 2—no improvement, 3—no obvious improvement, 4—obvious improvement, and 5—super improvement. (c) The level of cueing to overcome FoG: 1—absolutely no improvement, 2—no improvement, 3—no obvious improvement, 4—obvious improvement, and 5—super improvement. (d) The trigger speed of cueing is fast enough? (e) How to improve mobility by cueing? A—cueing can prevent FoG appears, B—cueing can reduce times/duration of FoG appears, C—cueing can overcome FoG, and D—no improvement.

A total of 16 subjects participated in the second-phase clinical trials and took a questionnaire survey at the end of the trials. A total of 11 subjects experienced FoG during the trials (a total of 623 times) when the IWS worked at the automatic cueing mode. The questionnaire results (Figure 6a) indicate that 88% of subjects considered their mobility was enhanced (Figure 6b and Supplementary Figure S8A), 70% thought IWS could help to overcome FoG (Figure 6c). Totally, 82% of patients thought that the response time of sensory cueing was enough upon the occurrence of FoG (Figure 6d). Totally, 81% of patients felt that the IWS was comfortable and practical. Figure 6e indicates that 13% of the subjects confirmed that the IWS can prevent FoG occurrence, 56% thought that IWS could reduce the number of FoG occurrences, 25% of them thought that IWS could help them overcome FoG. Moreover, as individual variation in gait was large, the threshold values of 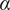 and 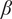 utilized for FoG detection should be changed in the APP manually according to the subject’s walking styles and customer habits.

Approximately half of the subjects selected the auditory device and the other half chose laser lights, respectively, as their preferred choice (Supplementary Figure S8B). More results of questionnaire survey are shown in Supplementary Figures S9 and S10.

## Discussion

### Benchmarking

Accelerometers or inertial measurement units (IMUs) attached to lower limbs have been common due to low cost, lightweight, ease of use, and durability. Because of the presence of a joint with the increased degrees of freedom, the data obtained become unnecessarily complex and the important features of very disordered gaits are masked. In the contrast, plantar pressure sensors or switches can directly measure the reacting force between the feet and the ground, which is more efficient to detect disordered gaits. But most pressure sensors or switches have limited fatigues resistance and cost. We have developed durable PSUs working beyond 1,000,000 cyclic compressions without breakdown. Moreover, after PUSs are embedded in insoles, the binary output can be achieved through the inner integrated circuit. Therefore, the PSUs have a greatly reduced quantity of output data compared to accelerometers or IMUs. Subsequently, there are no needs for filters and the Fourier transfer from the time to the frequency domain. And so, a simple but efficient algorithm has been dedicated to FoG detection. Meanwhile, PSUs can accurately distinguish the important events of FoG, and the accuracy is up to ~95%.

The IWS has the following features, as listed in Table 2. Our study has the largest number of FoG occurrences as compared with others. First, the top performance has been achieved in terms of sensitivity and specificity, 3% higher than the second-best from a split dataset (Mikos et al., 2019), also slightly higher than the second-best from a completed dataset (Lorenzi et al., 2016; Kita et al., 2017). Secondly, the sensing network for gait monitoring is embedded in insoles. There are no additional accessories compared to those with accelerometers or IMU fixed on the ankle, shin, hip, waist, or others (Bächlin et al., 2010; Mazilu et al., 2012; Kim et al., 2015; Pepa et al., 2015; Lorenzi et al., 2016; Ahn et al., 2017; Kita et al., 2017; Punin et al., 2017; Mikos et al., 2019). Thirdly, multisensory cueing functions are provided in IWS, including auditory and visual cueing, with the widest choices for intervention. Fourthly, a smartphone is utilized to conduct the gait feature extraction and classification, to deliver the control signals to cueing devices (Mazilu et al., 2012; Kim et al., 2015; Pepa et al., 2015; Kita et al., 2017; Punin et al., 2017). Compared to others’ works, we go further and have developed an APP for the IWS, which has been used in clinical trials. Fifthly, the IWS deals with a much smaller amount of data from the sensing insoles, thus the fast calculation for real-time FoG detection is achieved in comparison to the accelerometers or IMU-related methods. For example, Lorenzi et al.’s (2016) work was based on a time-domain analysis of the sensor signals. The raw signals of the accelerometer and gyroscope are fused using an orientation filter applicable to IMUs consisting of tri-axis gyroscopes and accelerometers. Their algorithm allows to achieve a high precision but paid in terms of the number of calculations concerning the low pass filtering and the root mean square method. Similarly, Mikos et al.’s (2019) work demonstrates an machine learning-based classification algorithm and achieves a high precision, which is also paid in terms of the number of calculations. Therefore, compared with various proposed FoG detection systems in literature, this wearable system has merits of more selection of sensory cueing methods, application of smart cell phone, high sensitivity, and specificity under split data set.

**Table 2.** Comparison of various wearable FoG detection systems in the literature

	Sensor				Algorithm		Data		Performance (%)			Split
Work (ref. no.)	Type	Location	Cues	CP	ML	Nf	Subjects	FoG	Sens	Spec	Accu	
Bächlin et al., 2010	Acc	Multi	A	×	×	2	10	237	73.1	81.6	—	×
Mazilu et al., 2012	Acc	Multi	A, S	√	√	7	10	237	62.1	95.2	—	√
Pepa et al., 2015	Acc	Waist	A	√	×	3	18	73	82.3	76.8	—	×
Kim et al., 2015	Acc	Multi	A, S	√	√	12	9	—	86.0	91.7	—	√
Lorenzi et al., 2016	IMUs	Shin	A	×	×	1	16	—	94.5	96.7	95.6	×
Ahn et al., 2017	IMUs	Glass	V	×	×	1	10	42	97.0	88.0	—	×
C. Punin et al., 2017	IMUs	Ankle	S	√	×	2	8	27	86.7	60.6	—	×
(Kita et al., 2017)	IMUs	Shin	A, S	√	×	1	32	—	97.6	93.4	—	×
Mikos et al., 2019	IMUs	Shin	A, S	×	√	3	63	485	95.6	90.2	—	√
This work	PSUs	Insoles	A, V	√	×	2	30	1,502	97.6	95.0	95.2	√

Abbreviations: *Sensor*, accelerometers (Acc), inertial measurement units (IMUs), pressure sensing units (PSUs). *Sensor Location*, the position of sensors, Multi means multiple sensors located chest, waist, knee, ankle, and so on. *Cues*, auditory (A), somatosensory (S), visual (V). *CP*, cellphone used to collect data and run algorithms. *Algorithm*, machine learning (ML), number of features employed (Nf). *Data*, total number of subjects (Subjects), the total number of FoG occurrence (FoG). *Performance*, sensitivity (Sens), specificity (Spec), Accuracy (Accu). The value of accuracy lies between the values of sensitivity and specificity. *Split*, Split the dataset into a training and testing set.

## Conclusions

In this study, an IWS, equipped with a pair of pressure-sensing smart insoles, a smartphone with a customer-made APP as well as wireless sensory cueing devices, has been developed for people with PD. The system can accurately detect abnormal gait in real-time with low power consumption, and provide timely sensory cueing within a short period. There are two unique key technologies in this system. The first is the novel PSUs embedded in the smart insoles. They are sensitive to the events of FS and FO, possessing the merits of lightweight, comfortable, seamless, and reliable integration as well as an excellent fatigue resistance of over 1,000,000 compression cycles. More importantly, the PSUs have a binary output thus greatly reducing the amount of data to be transferred wirelessly and processed, facilitating a simple and fast algorithm for the FoG detection. The other feature is the highly efficient algorithm dedicated to the binary outputs of PSUs.

In the clinical trials, the IWS demonstrated an accuracy of 94.1% in real-time FoG detection with a mean delay of 0.37 s from the onset of FoG to the cueing activation. The FoG occurrence reduced from 44 times/trial to 39 times/trial when the grouped PD subject walked with cueing in comparing to other groups walked without cueing. Through a subjective questionnaire of 16 PD patients, 88% of patients confirmed that this wearable system could effectively enhance their walking, 81% of patients regarded the system was comfortable to use, and 70% of patients overcame the FoG. Therefore, the IWS has a high sensitivity to gait phase events, a high detection accuracy of FoG, and a timely sensory cueing, which makes it an effective, powerful, and convenient tool for enhancing the mobility of people with PD.

## Data Availability

The main data supporting the results in this study are available within this article and its Supplementary Information. Additional data related to this article may be requested from the authors.

## Supplementary material

For supplementary material accompanying this paper visit https://doi.org/10.1017/wtc.2022.9. click here to view supplementary material
