# Supplementary material for: Intelligent wearable system with accurate detection of abnormal gait and timely cueing for mobility enhancement of people with Parkinson’s disease
Source: Wearable Technol. 2022 Jun 28;3:e12. doi: 10.1017/wtc.2022.9 (PMC10936378; doi:10.1017/wtc.2022.9)
Supplement: Supplementary file 1 [file wtcsup.zip › S2631717622000068sup001.docx]

Table 1: Logistic regression coefficients (with interaction terms) and selected features for each classifier model (Falls, BBS and MMSE) for each sensor location (Torso or Thigh). Separate models are created for each gender (M or F) as well as for both genders pooled (All). Falls models classify participants as faller or non-faller. Berg Balance Scale (BBS) models classifying each participants balance as impaired or unimpaired, while Mini-Mental State Examination (MMSE) models classify each participant as cognitively impaired or cognitively intact. Beta refers to logistic regression coefficient for each of the ‘n’ features or interaction terms (the listed coefficients are in the following order, each feature (in order 1, 2, ...,n), constant term, the interaction terms (pairwise products of the features in order (1, 2), (1, 3), ..., (1,n), (2, 3), ..., (n–1,n))).

| **Torso Falls** | | | | | | | |
| --- | --- | --- | --- | --- | --- | --- | --- |
| **Beta (All)** | **Features (All)** | **Beta (M)** | | **Features (M)** | | **Beta (F)** | **Features (F)** |
| -0.28551 | TotalTime | -262.375 | | TotalTime | | -0.45542 | TotalTime |
| 0.118723 | MeanSSSTime | -112.768 | | RMS_X_Total_Acc | | 0.142 | MeanSSSTime |
| -1.79124 | MeanStandTime | -591.451 | | H_Acc | | -0.49076 | RMS_Y_Total_AV |
| 1.250259 | RMS_Y_Total_Acc | -62.4031 | | Fmed_AngVel | | -0.58182 | H_AngVel |
| 0.063962 | H_AngVel | -303.027 | | AV_X_stand_start_std | | 0.087825 |  |
| -1.09173 | AccZ_stand_start_std | 167.3093 | |  | | 0.626301 |  |
| 0.952618 |  | -335.076 | |  | | -1.63697 |  |
| -1.54489 |  | -74.836 | |  | | -0.59945 |  |
| 0.674276 |  | 61.20151 | |  | | 1.962895 |  |
| -1.26409 |  | 21.9156 | |  | | 0.120557 |  |
| 0.651943 |  | 71.69537 | |  | | -1.256 |  |
| -0.87162 |  | -824.694 | |  | |  |  |
| 0.589206 |  | 320.0747 | |  | |  |  |
| -5.23086 |  | 42.49934 | |  | |  |  |
| -3.22293 |  | 11.1571 | |  | |  |  |
| -2.26885 |  | 17.84547 | |  | |  |  |
| 3.549486 |  |  | |  | |  |  |
| 2.500645 |  |  | |  | |  |  |
| 4.555671 |  |  | |  | |  |  |
| -3.02818 |  |  | |  | |  |  |
| 1.917393 |  |  | |  | |  |  |
| -0.2245 |  |  | |  | |  |  |
| **Torso BBS** | | | | | | | |
| **Beta (All)** | **Features (All)** | **Beta (M)** | | **Features (M)** | | **Beta(F)** | **Features (F)** |
| -1.59430291 | TotalTime | -1.54493 | | TotalTime | | -0.78632 | TotalTime |
| 0.684688782 | CVSSSTime | -1.01681 | | Jerk_Y_Total_AV | | 0.951372 | AccZ_mid_stand_range |
| 2.258265423 | AccZ_sit_end_range | -1.51955 | |  | | 0.515303 |  |
| -0.61288027 | Age | -2.84113 | |  | | 0.931593 |  |
| 0.502962204 | Height |  | |  | |  |  |
| -0.48733141 |  |  | |  | |  |  |
| 0.643418667 |  |  | |  | |  |  |
| 1.049988596 |  |  | |  | |  |  |
| -1.12328742 |  |  | |  | |  |  |
| -0.73067677 |  |  | |  | |  |  |
| -0.28190906 |  |  | |  | |  |  |
| -0.72037164 |  |  | |  | |  |  |
| 0.601329637 |  |  | |  | |  |  |
| 0.520758893 |  |  | |  | |  |  |
| -0.0102241 |  |  | |  | |  |  |
| -0.88400988 |  |  | |  | |  |  |
| **Torso MMSE** | | | | | | | |
| **Beta (All)** | **Features (All)** | **Beta (M)** | **Features (M)** | | | **Beta (F)** | **Features (F)** |
| -1.19768 | TotalTime | -1.75571 | TotalTime | | | -1.48733 | TotalTime |
| 0.210547 | MeanStandTime | 1.813448 | MeanSSSTime | | | -0.63425 | NumReps |
| 0.308841 | JerkMLTotal_Acc | 1.383253 | MeanStandTime | | | 0.198885 | AV_Y_sit_end_range |
| 3.791304 | Gender | -1.7603 | H_AngVel | | | 0.198847 | Height |
| -1.25175 | Height | -0.39048 |  | | | -1.2828 |  |
| -1.64531 |  | 2.811687 |  | | | 0.48077 |  |
| -0.41792 |  | -4.49996 |  | | | -1.06389 |  |
| 1.396462 |  | -0.96186 |  | | | -1.37736 |  |
| 0.198001 |  | 4.464427 |  | | | 0.416628 |  |
| 0.840082 |  | 1.947501 |  | | | 1.462153 |  |
| -1.62111 |  | -6.54895 |  | | | -0.40434 |  |
| -0.65657 |  |  |  | | |  |  |
| -0.78265 |  |  |  | | |  |  |
| -3.22155 |  |  |  | | |  |  |
| -0.25002 |  |  |  | | |  |  |
| -0.60114 |  |  |  | | |  |  |
| **Thigh falls** | | | | | | | |
| **Beta (All)** | **Features (All)** | **Beta (M)** | **Features (M)** | | | **Beta (F)** | **Features (F)** |
| -1.08608 | TotalTime | 6.822981 | TotalTime | | | 0.278401 | TotalTime |
| -0.95306 | MeanSSSTime | 8.352301 | MeanStandTime | | | 0.415609 | NumReps |
| 0.467276 | MeanStandTime | 21.5753 | SEF_Acc | | | -0.58565 | Fmed_Acc |
| -4.7433 | AV_Z_stand_start_mean | 21.25585 | AV_Z_mid_stand_std | | | 1.162062 | AV_Z_sit_end_mean |
| -0.06908 |  | -7.94483 |  | | | -1.04566 |  |
| 2.6513 |  | 46.16662 |  | | | 2.295257 |  |
| -7.35278 |  | 0.909128 |  | | | 0.165961 |  |
| 0.527251 |  | -0.31508 |  | | | -0.24387 |  |
| 6.425441 |  | 96.67009 |  | | | -1.31447 |  |
| -0.56781 |  | -62.5886 |  | | | -3.16814 |  |
| 2.431397 |  | -7.96196 |  | | | 1.26098 |  |
| **Thigh BBS** | | | | | | | |
| **Beta (All)** | **Features (All)** | **Beta (M)** | | **Features (M)** | | **Beta (F)** | **Features (F)** |
| -1.12168 | TotalTime | -3.99345 | | TotalTime | | -0.89449 | TotalTime |
| 0.762442 | MeanStandTime | 25.5061 | | CV_SitTime | | 1.023556 | CVSSSTime |
| 0.870259 | H_AngVel | 15.32421 | |  | | 0.062955 | Age |
| 0.224389 | AccZ_stand_start_range | -162.348 | |  | | 1.264003 | Weight |
| -0.31987 |  |  | |  | | -0.61986 |  |
| -0.38118 |  |  | |  | | 0.73522 |  |
| -0.22104 |  |  | |  | | -0.46801 |  |
| -0.16759 |  |  | |  | | 0.381555 |  |
| 2.433635 |  |  | |  | | -0.13175 |  |
| -1.83125 |  |  | |  | | -1.70983 |  |
| -0.21487 |  |  | |  | | 0.054366 |  |
| **Thigh MMSE** | | | | | | | |
| **Beta (All)** | **Features (All)** | **Beta (M)** | | **Features (M)** | **Beta (F)** | | **Features (F)** |
| -0.73574 | TotalTime | -0.82139 | | TotalTime | -0.85046 | | TotalTime |
| 0.161819 | CVSSSTime | 0.026681 | | AccZ_stand_start_range | 0.129485 | |  |
| -0.08371 | MeanSitTime | -1.85174 | |  |  | |  |
| -0.1137 |  | -2.52003 | |  |  | |  |
| 0.029561 |  |  | |  |  | |  |
| -0.16808 |  |  | |  |  | |  |
| 0.314284 |  |  | |  |  | |  |
