# Supplementary material for: Intelligent wearable system with accurate detection of abnormal gait and timely cueing for mobility enhancement of people with Parkinson’s disease
Source: Wearable Technol. 2022 Jun 28;3:e12. doi: 10.1017/wtc.2022.9 (PMC10936378; doi:10.1017/wtc.2022.9)
Supplement: Supplementary file 1 [file wtcsup.zip › S2631717622000093sup001.docx]

**Supplementary information**

**Intelligent wearable system with accurate detection of abnormal gait and timely cueing for mobility enhancement of people with Parkinson’s Disease**

Bao Yang,^1,2^ Ying Li^1,4^ Fei Wang,^1,6^ Stephanie Auyeung,^1,5^ Manyui Leung,^1,3^ Margaret Mak, ^1,5^ and Xiaoming Tao^1,3,4*^

^1^ Research Institute for Intelligent Wearable Systems, The Hong Kong Polytechnic University, Hong Kong, China

^2^ School of Civil Engineering and Transportation, South China University of Technology, Guangzhou, China

^3^ Institute of Textile and Clothing, The Hong Kong Polytechnic University, Hong Kong, China

^4^ The Hong Kong Polytechnic University Shenzhen Research Institute, Shenzhen, China

^5^ Department of Rehabilitation Sciences, The Hong Kong Polytechnic University, Hong Kong, China

^6^ School of Textile Materials and Engineering, Wuyi University, Jiangmen, China

^*^Corresponding author: xiao-ming.tao@polyu.edu.hk

**Supplementary Information**

**Supplementary Information contains：**

**Supplementary Texts：**

S1. Gait parameters

S2. Questionnaire

S3. Definition of sensitivity, specificity, and accuracy

**Supplementary Figures: Fig. S1 - Fig.S9**

**Supplementary Tables：Table S1 – Table S6**

**Description of Supplementary Videos: Mov.S1 - Mov.S2**

**Supplementary Texts：**

**S1. Gait parameters**

Once the events of FS and FO are detected, the peak duration of the $i^{th}$ single support ($P_{Ss}$) and swing ($P_{Sw}$) can be given by the following equations.

$P_{Ss}(i)=F_{O}(i+1)-F_{S}(i),for i=1,2,\cdots,m$ (S1)

$P_{Sw}(i)=F_{S}(i)-F_{O}(i),for i=1,2,\cdots,n$ (S2)

where $F_{S}(i)$ and $F_{O}(i)$ represent the moment of the $i^{th}$FS and FO, respectively. $m$and *n* represent the total number of stances and swings, respectively.

Double support is defined as that both feet are on the ground for support. Each double support begins at FS and ends at FO. And thus, the peak of the peak duration for the $i^{th}$double support (*P_DDS*) can be given by

$P_{DS}(i)=F_{E}(i)-F_{B}(i), for i=1,2,\cdots,k$ (S3)

where $F_{B}(i)$ and $F_{E}(i)$ represent the beginning and end of the $i^{th}$ double support, respectively. And $k$ is the total number of double supports.

**S2. Questionnaire**

A 17- item questionnaire on intelligent wearable systems was distributed to 16 subjects in the wear trials. The questionnaire includes three parts: 6 items on comfortability and practicality, 6 items on assessment of cueing, and 5 items on suggestions for improvement. The items in the questionnaire are listed below.

Questions & answer or 5-point scale (1 - Strongly Disagree, 2 -Disagree, 3 - Nurture, 4 - Agree, 5 - Strongly Agree)

**Part 1: Comfortability and practicality of IWS**

1) Smart insoles are comfortable.

2) The position of laser light is comfortable.

3) The weight of laser light is acceptable.

4) The whole set system is easy to wear.

5) APP is easy to use.

**Part 2: Assessment o cueing**

1) Preferred cueing of the ISW

2) Whether can the system improve mobility?

3) The level of improvement of walking.

4) The level of the cueing to overcome FoG.

5) The trigger speed of cueing is fast enough.

6) How to improve your mobility by cueing?

**Part 3: Suggestions for improvement**

1) Environment of the usage.

2) Did you use other cueing that is effective for you?

3) Which kind of cueing is effective for you?

4) If the somatosensory cue is integrated into the ISW, do you willing to try?

5) Which method do you prefer to cancel the cueing if it bothers you?

6) Do you have any comments for the ISW?

**S3. Definition of sensitivity, specificity, and accuracy**

Sensitivity and specificity are statistical measures of the performance of a binary classification test, which were chosen to evaluate the performance of FoG detection like the references(Lorenzi et al., 2016; Mikos et al., 2019). First, some items should be defined. For example, if a step is detected as FoG by the proposed model and by the observers, its corresponding data are considered as true positive ($TP$). If a step is detected as non-FoG by the proposed model and by the observers, its corresponding gait data are considered true negative ($TN$). If a step is detected as FoG by the proposed model, while this step is detected as non-FoG by the observers, the corresponding gait data are considered false positive ($FP$). And if a step is detected as non-FoG by the proposed model, while this step is detected as FoG by the observers, the corresponding gait data are considered false negative ($FN$). *TP* and *TN* indicate that the result detected by the proposed model is consistent with that of the actual condition. Reversely, *FP* and *FN* indicate that the result detected by the proposed model is opposite to the actual condition. After the data points of gait were classified into different datasets of $TP$, $FP$, $TN$, and $FN$, the sensitivity, specificity and accuracy can be given by

$Sensitivity=TP/\left( TP+FN \right)$ (S5)

$Specificity=TN/\left( TN+FP \right)$ (S6)

$Accuracy=\left( TP+TN \right)/\left( TP+FP+TN+FN \right)$ (S7)

**Supplementary Figures:**


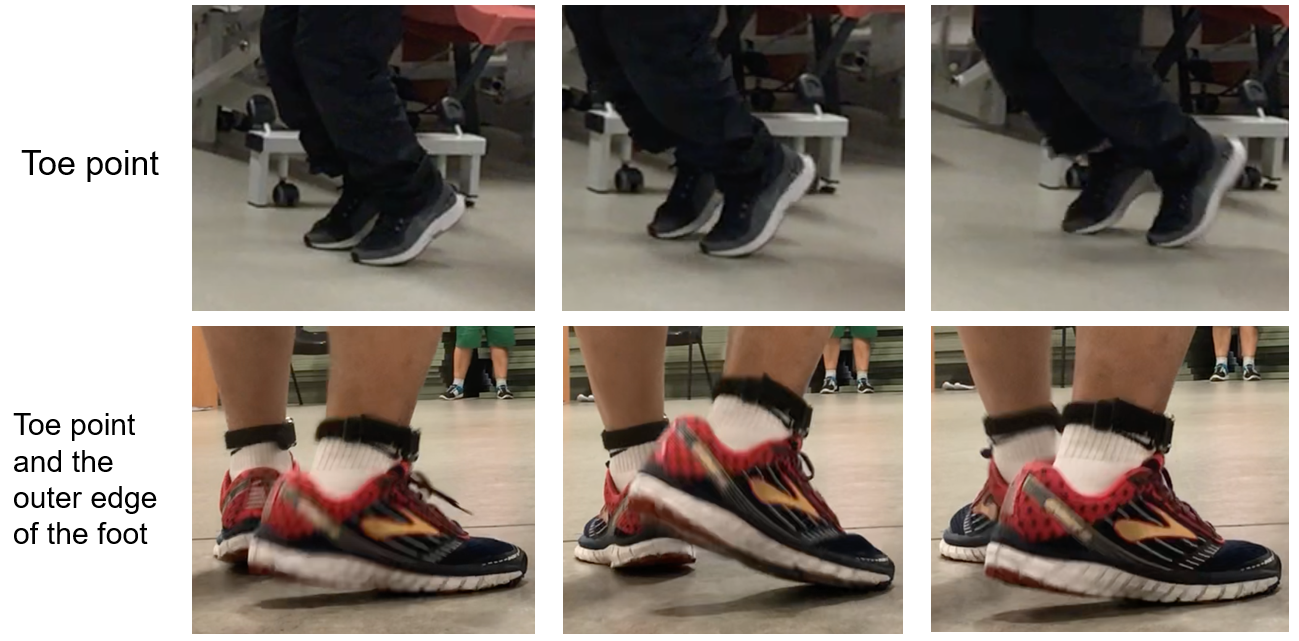


**Fig.S1** ǀ **Illustration of degenerate gaits of PD patients**. These photos captured in the trials show that the toe and the outer edge of the forefoot can be the main region for supporting.


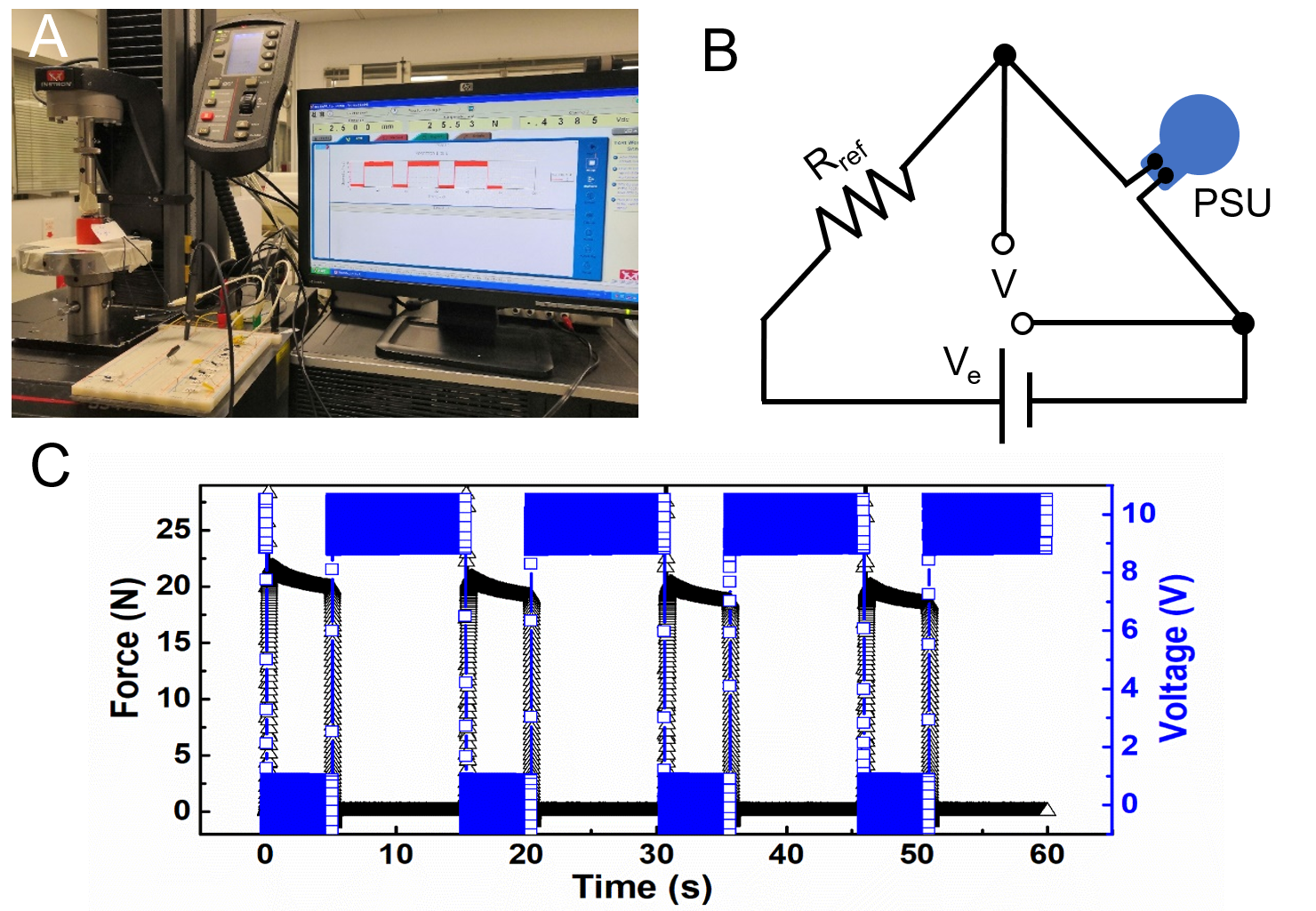


**Fig.S2 ǀ** **Illustration of the response time of bare PSUs under cyclic compression.** These tests were performed on a material machine (Series 5944, Instron®, America) installed with a load cell and a coupled voltage divider circuit. The applied force and the resistance change of the bare PSUs were recorded simultaneously. **A**, Physical photo of the experimental setup. **B**, Schematic of a voltage divider circuit. The loading and unloading speed are same as 35 mm/s. And a sampling rate of 1 kHz was chosen so that it is enough high to capture the sharp changes of the applied force and the divided voltage across the bare PSU. **C**, Illustrated output signals of the applied force and the particle voltage across the PSU. Here, the designed threshold pressure of the PSU is about 20 kPa. And enlarged curves are shown in **Fig.**2b.


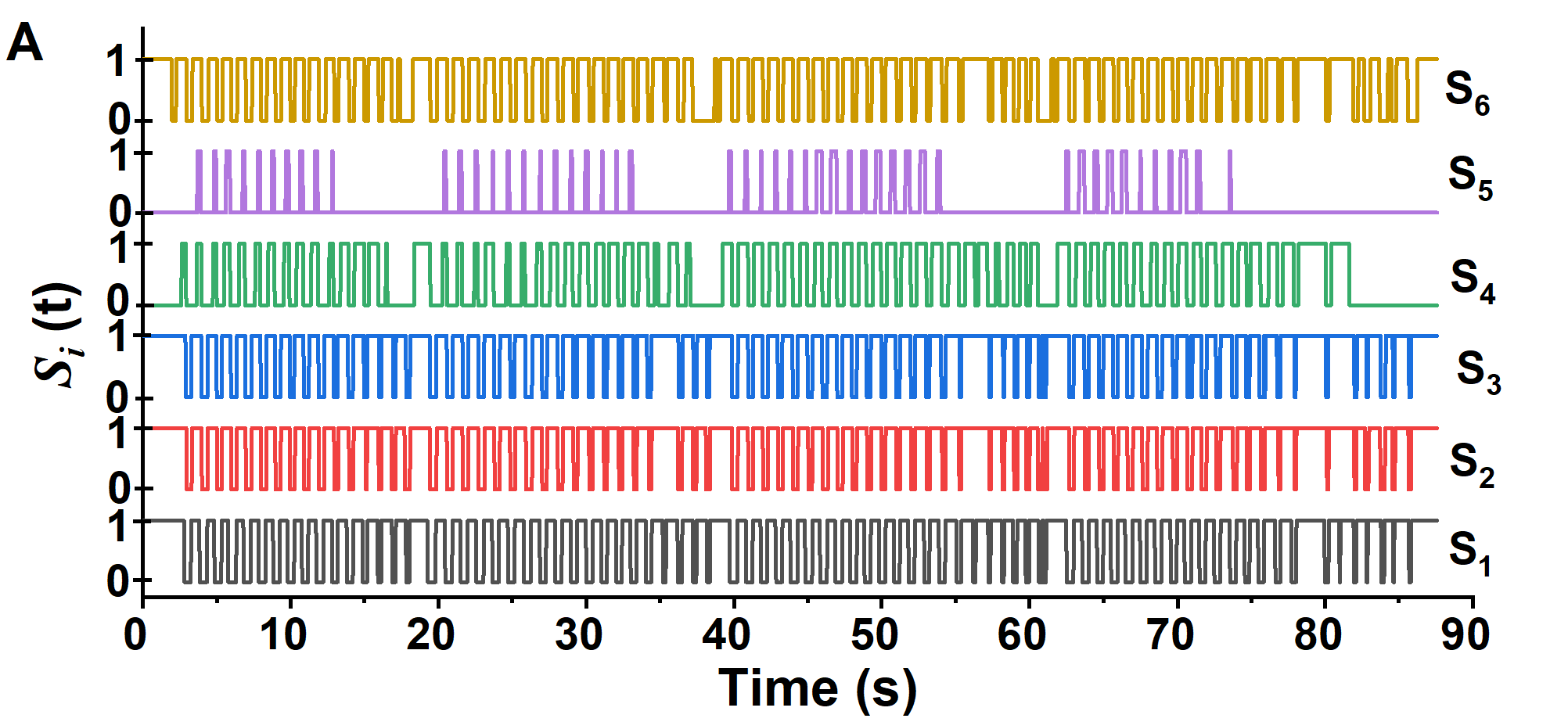


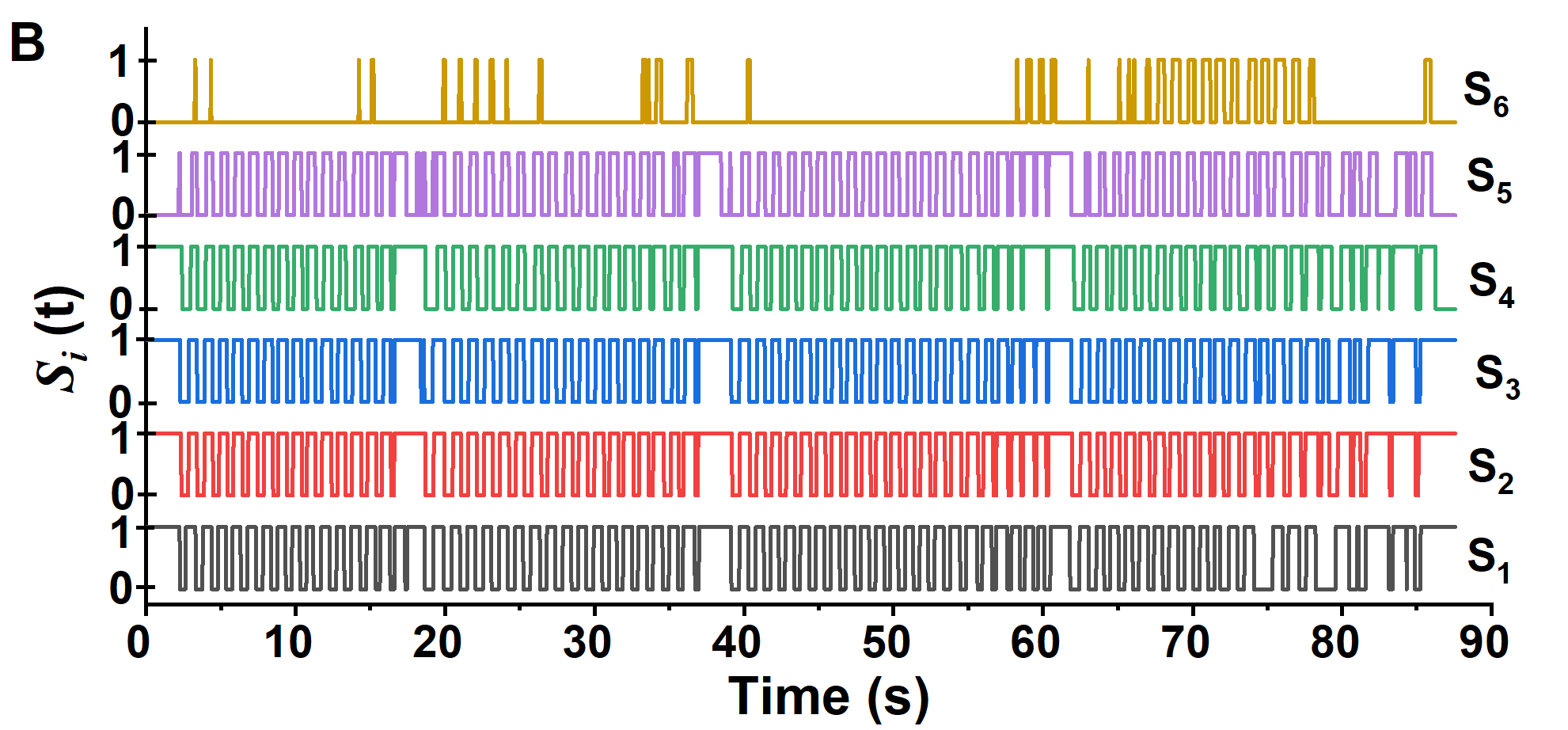


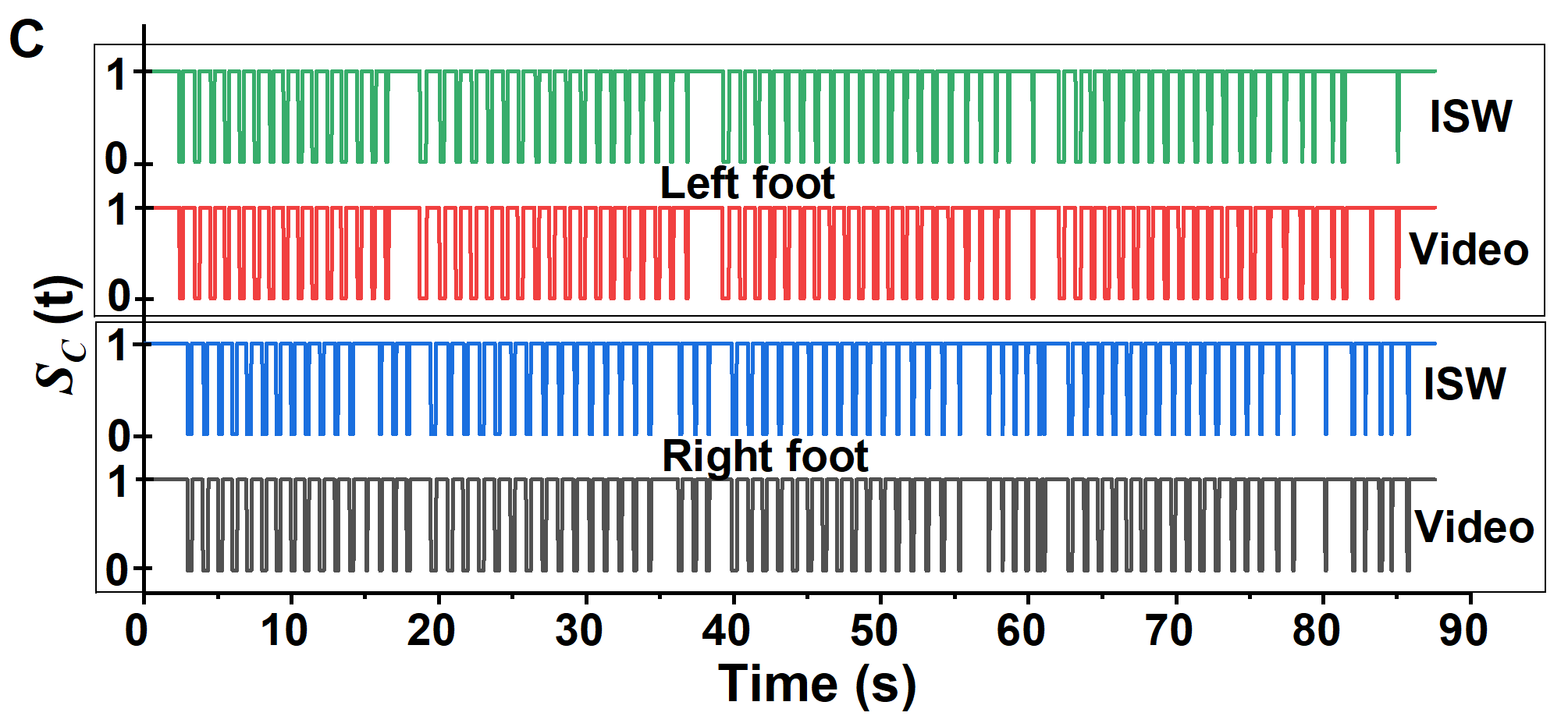


**Fig. S3 ǀ Comparison between the collected data from ISW and video shorts. A**, Illustrated signals of the left insole. **B**, Illustrated signals of the right insole. S_1_, S_2_, S_3_, S_4_, S_5_ and S_6_ are the data collected from corresponding PSU in Fig.1c. The subject had 62 kg in weight, the height of 170 cm, age of 79 years old, the shoe size of 42.0, and H & Y stage of 1.5. The raw signals of each PSU with the sampling rate of 32Hz are listed. **C**, Combined signals of each insole versus video observation as benchmarks, showing that the accuracy of ISW is over 95% for each foot. Here, the gait phase of stance and swing were detected by professionals and expressed as “1” and “0”, respectively.











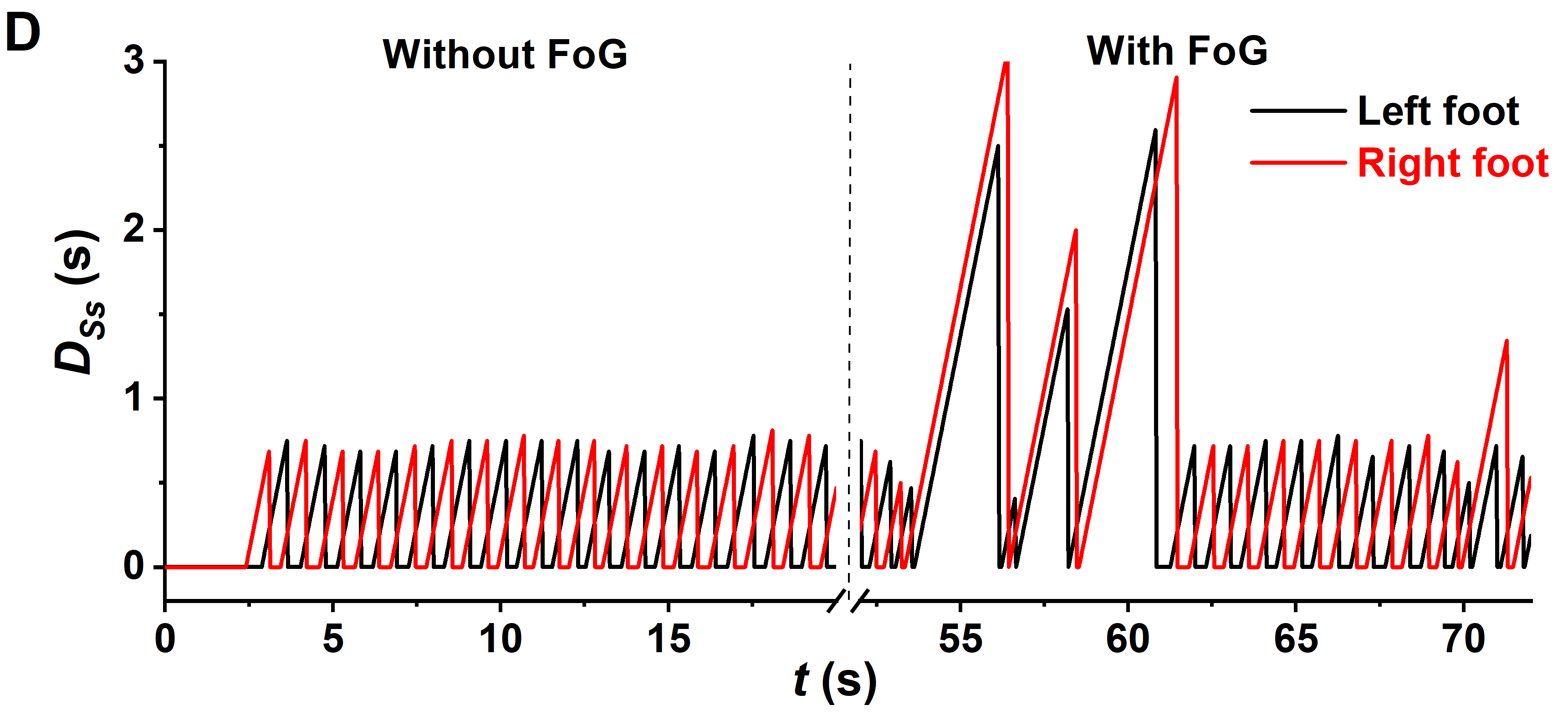








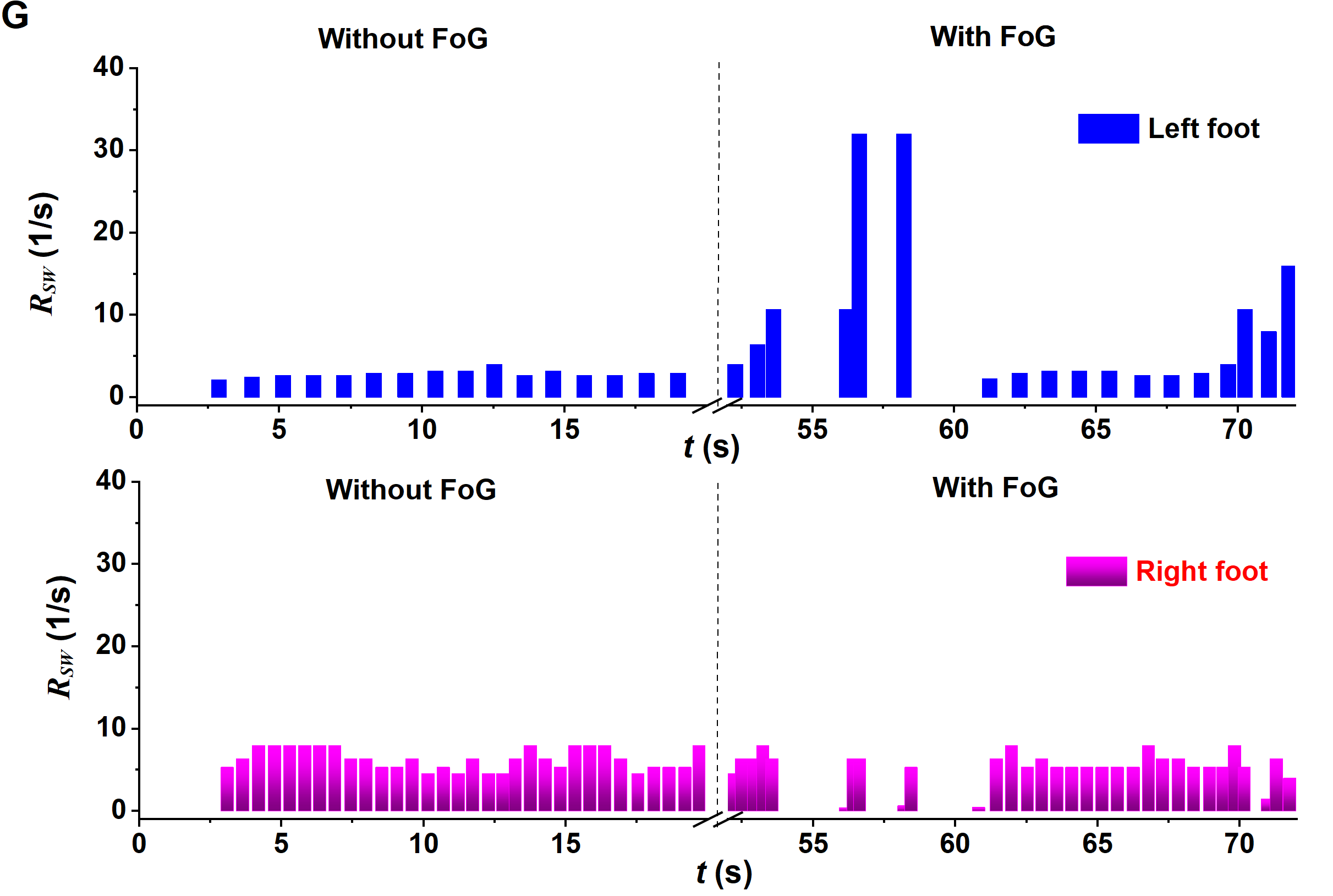


**Fig. S4 ǀ PD subject walked without and with small shuffling steps. A**, PSU signals of the left insole. **B**, PSU signals of the right foot. **C**, Comparison between corresponding results of IWS and those of video shots. **D-F**, Corresponding duration of stance, double support, and swing, respectively. **G**, Corresponding reciprocal of $R_{Sw}$. The subject had the weight of 74 kg, the height of 187 cm, the age of 69 years old, the shoe size of 41.0, and H & Y stage of 3.0.


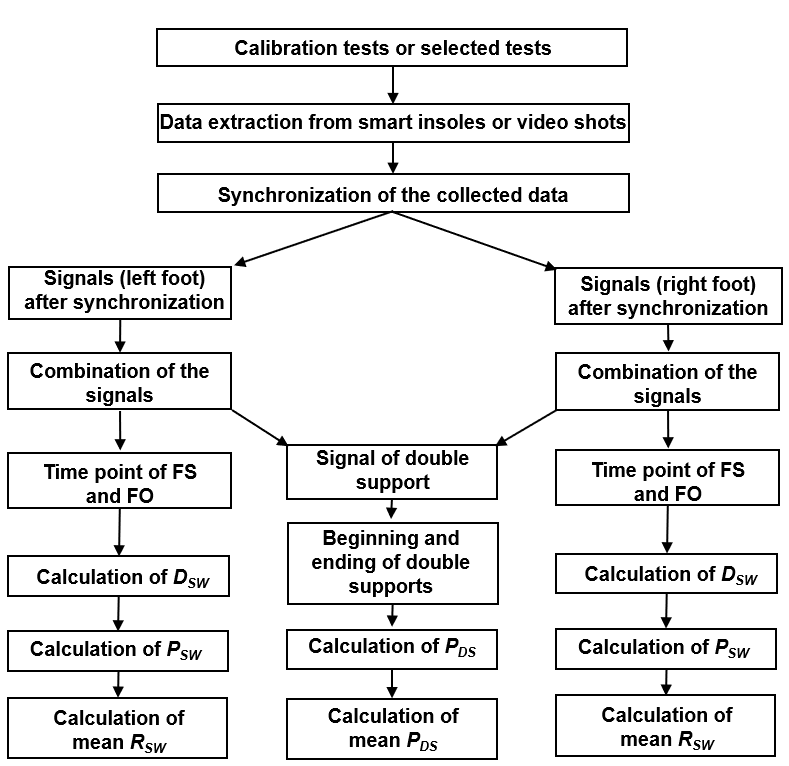


**Fig. S5 ǀ** Data flow of calibration tests. The calibration test was utilized to provide the mean value of the peak duration of double support ($\bar{P_{DS}}$) *and* the reciprocal of the mean value of the peak duration of swing ($\bar{R_{Sw}}=1/{\bar{P_{Sw}}}$), which represents the mobility of individual. It is noted that there are no FoG during the calibration tests. If one subject walked with FoG in all tests, the calibration results will be replaced by nominal values (0.22s for double support, 2.53 1/s and 2.50 1/s as RDSW for the left foot and right foot, respectively).


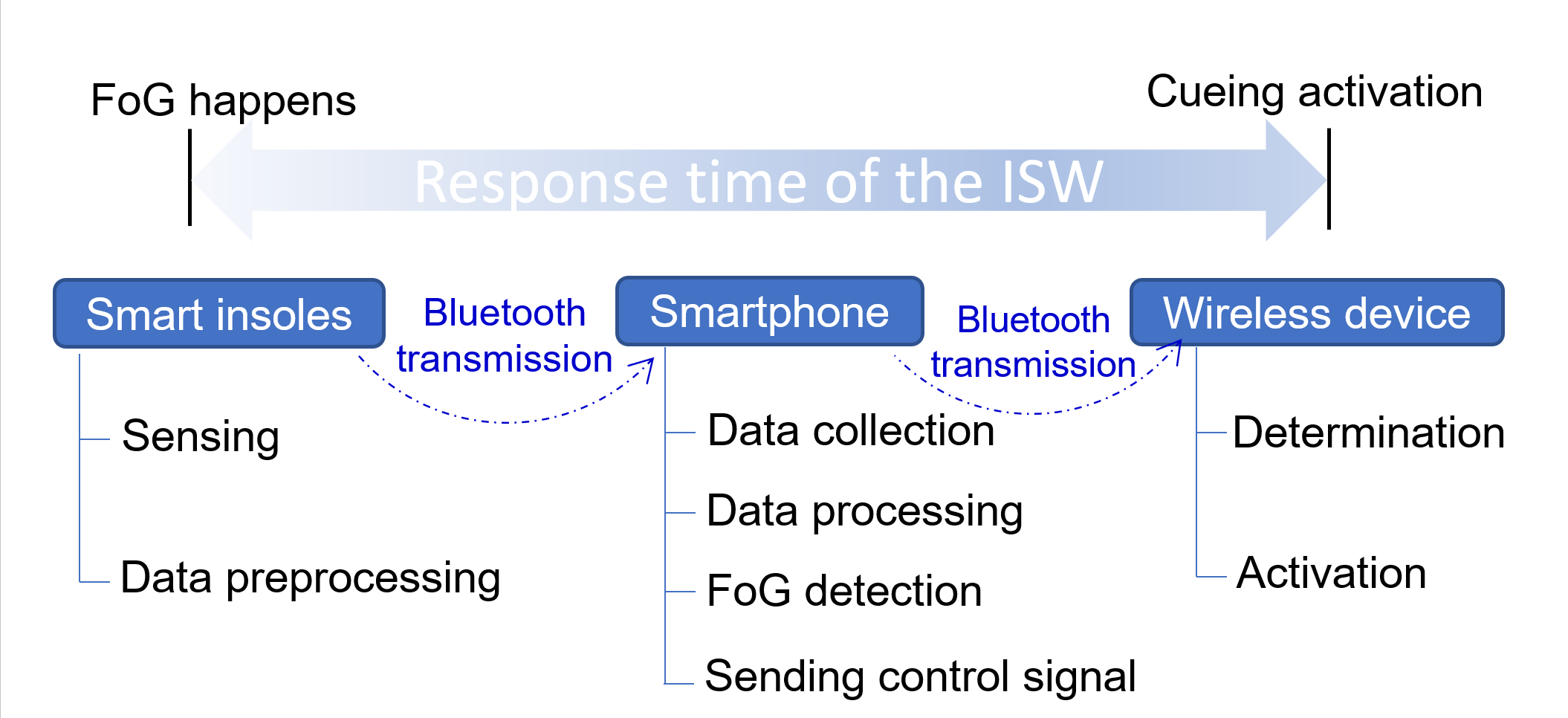


**Fig. S6 ǀ** **Schematic of the response time from the onset of FoG to provision of cueing,** including the time for sensing and data preprocessing in smart insoles, data transmission from smart insoles to the smartphone, data collection, data processing, FoG detection and sending control signal using smartphone, data transmission from smartphone to cueing devices, and determination and activation of the cueing devices.


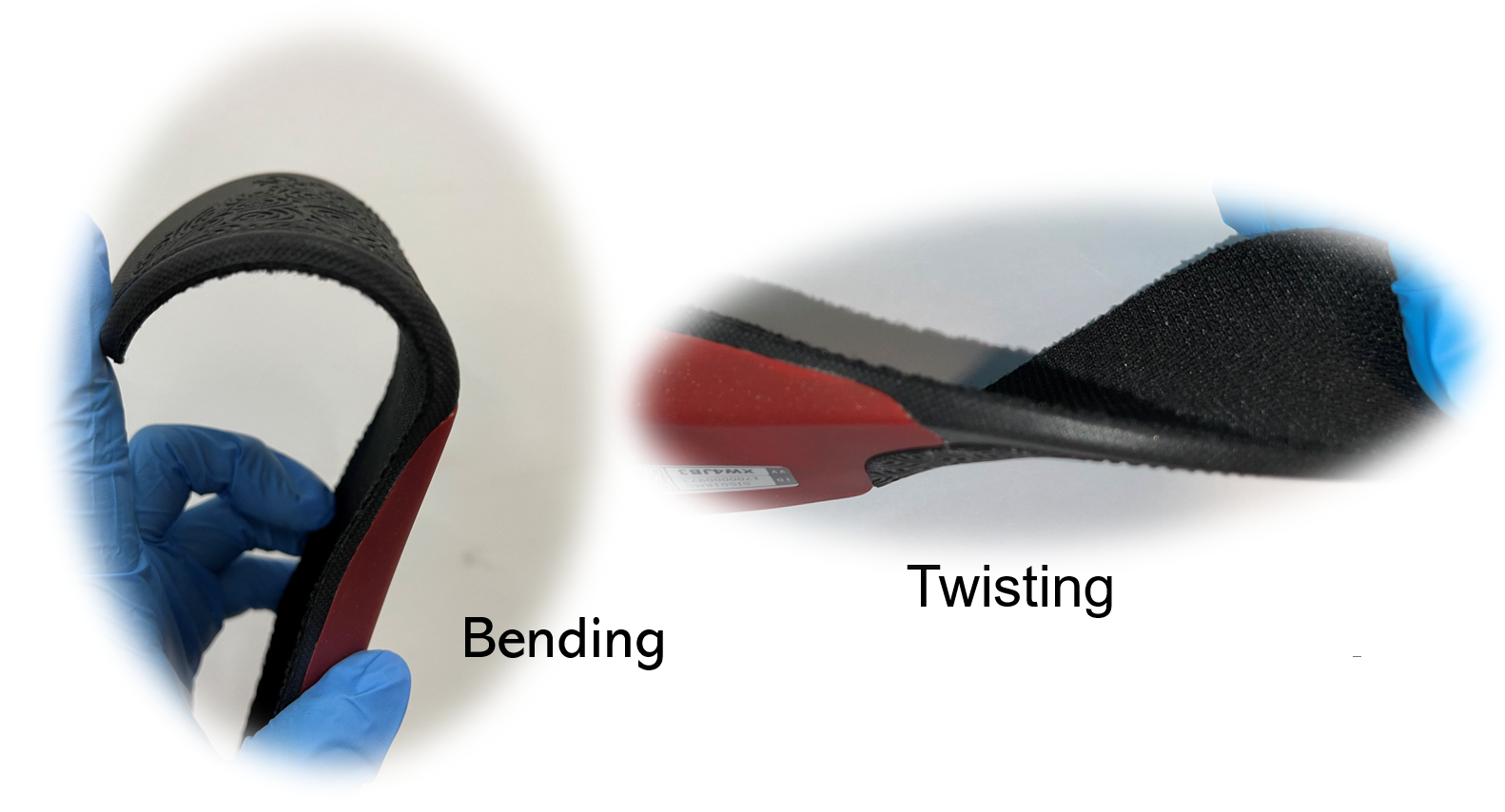


**Figure S7ǀ**Physical photo of smart insoles under bending and twisting


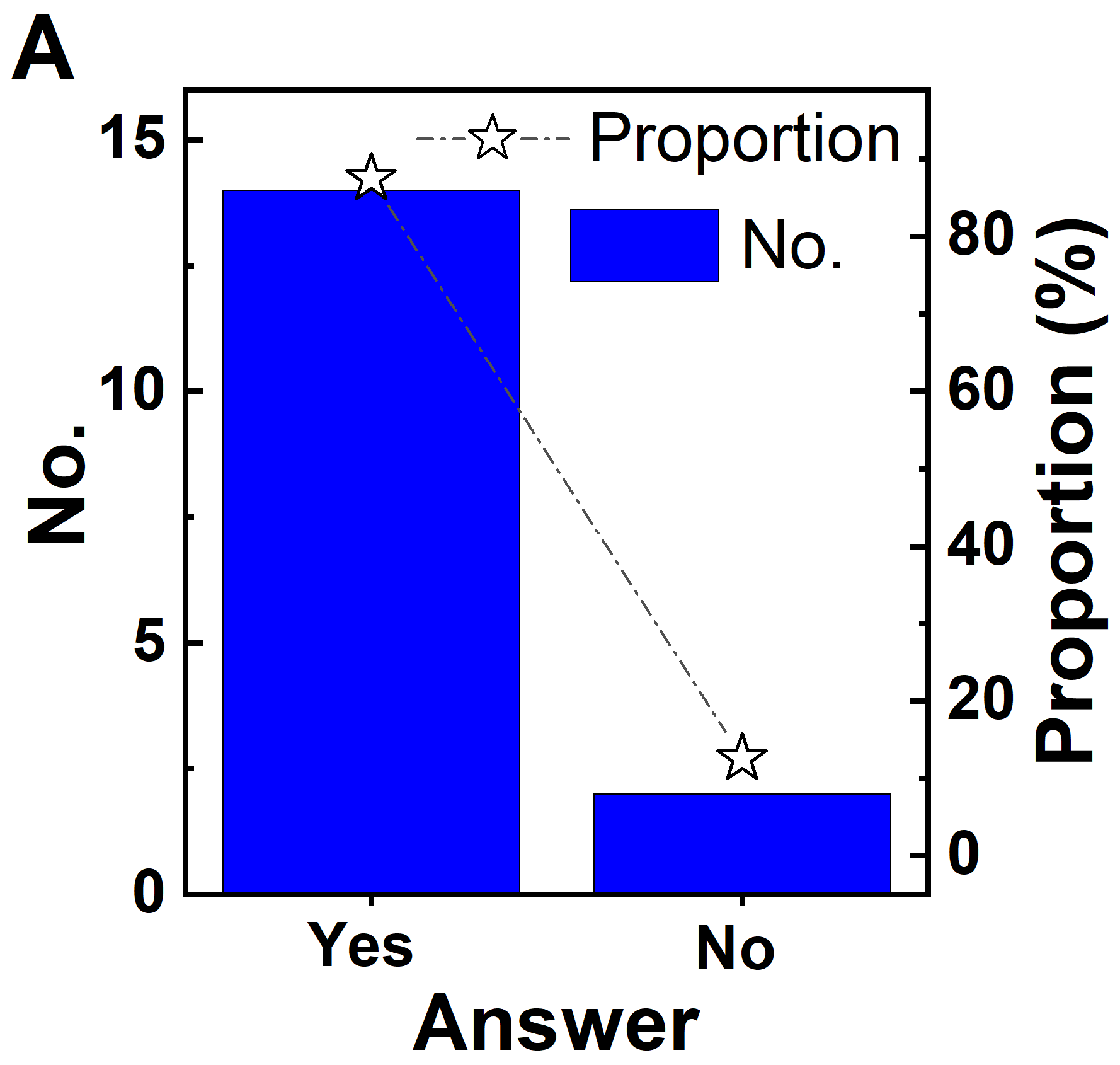

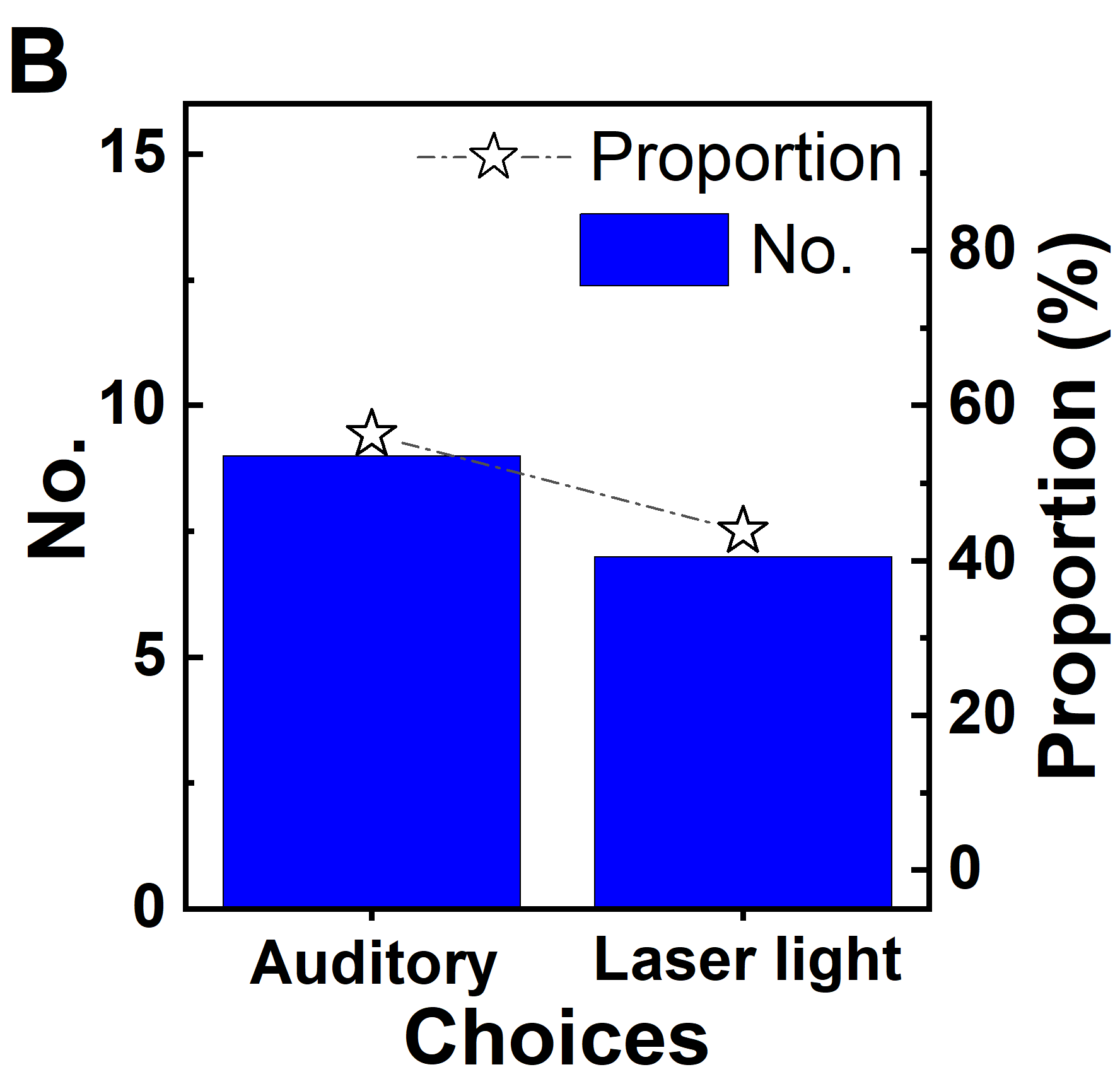


**Fig. S8 ǀ Questionnaire results of assessment of cueing**: **A**, Whether can the system improve the mobility? **B**, Preferred cueing.

**Results of questionnaire survey & user feedback.** As shown in **Fig.** S9A of Supplementary Information, about 75 % of the subjects, scoring at 4 and 5, think that the smart insoles are comfortable. Others, scoring lower than 4, are purposely induced by wearing two insoles in one shoe or wearing mismatch insoles during trails, respectively. 94 % of subjects, scoring at 4 and 5, indicate that the laser light cue devices are comfortable (Supplementary **Fig.** S9B) and their weight is acceptable (Supplementary **Fig.** S9C). 69 % of subjects, scoring at 4 and 5, think that the whole wearable system is easy to use (Supplementary **Fig.** S9D). Moreover, though most of the subjects rarely used other APPs than communication APPs like WeChat or WhatsApp, 75 % of them confirm that the developed APP is easy to use (Supplementary **Fig.** S9E). As 5 devices are required to connect via Bluetooth to the cellphone before the wearable system works, some 25 % of them, scoring at 3, thought that the connection of Bluetooth is too complicated. In general, the hardware of the IWS is comfortable and easy to use. Moreover, 94 % of the subjects would like to apply the wearable system in the environment of indoor and outdoor (Supplementary **Fig.** S10A). Among them, 25 % have experience of using other cueing devices of laser walking sticks, and 75 % have not utilized any visual and auditory cues before (Supplementary **Fig.** S10B). 56 % of subjects prefer auditory (Supplementary **Fig.** S10C) may be due to better privacy than laser lights and higher sensitivity than haptic cues. Finally, 50 % of the subjects prefer that the cueing doesn’t need to switch off manually after overcoming FoG or false trigger because it is acceptable to have about 30 s cueing. 25 % of the subjects would like to switch off the cueing by the special movement of feet. 13 % would switch off the cueing device by APP manually and double-tap the Bluetooth devices, respectively (Supplementary **Fig.** S10D).


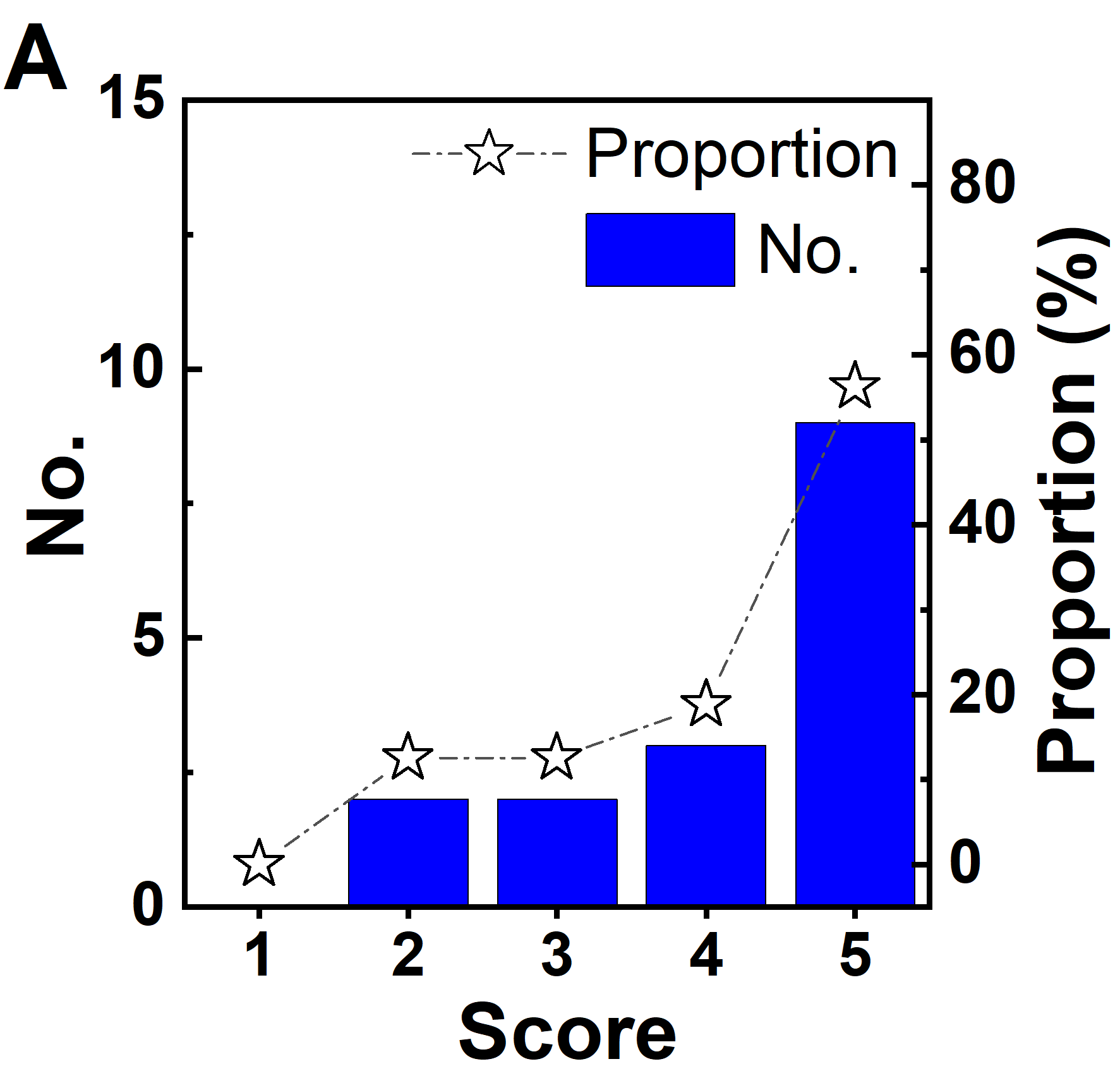

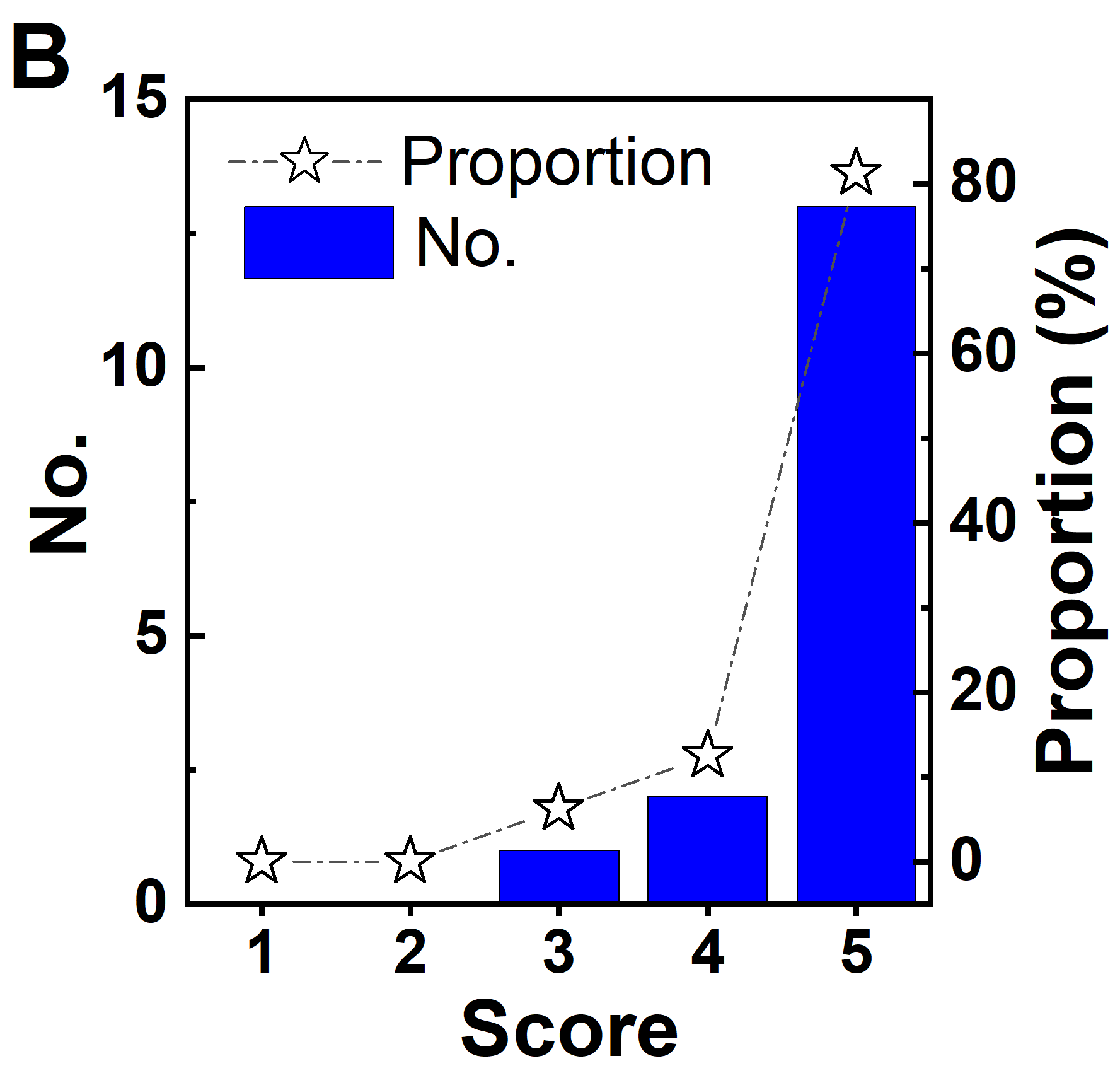


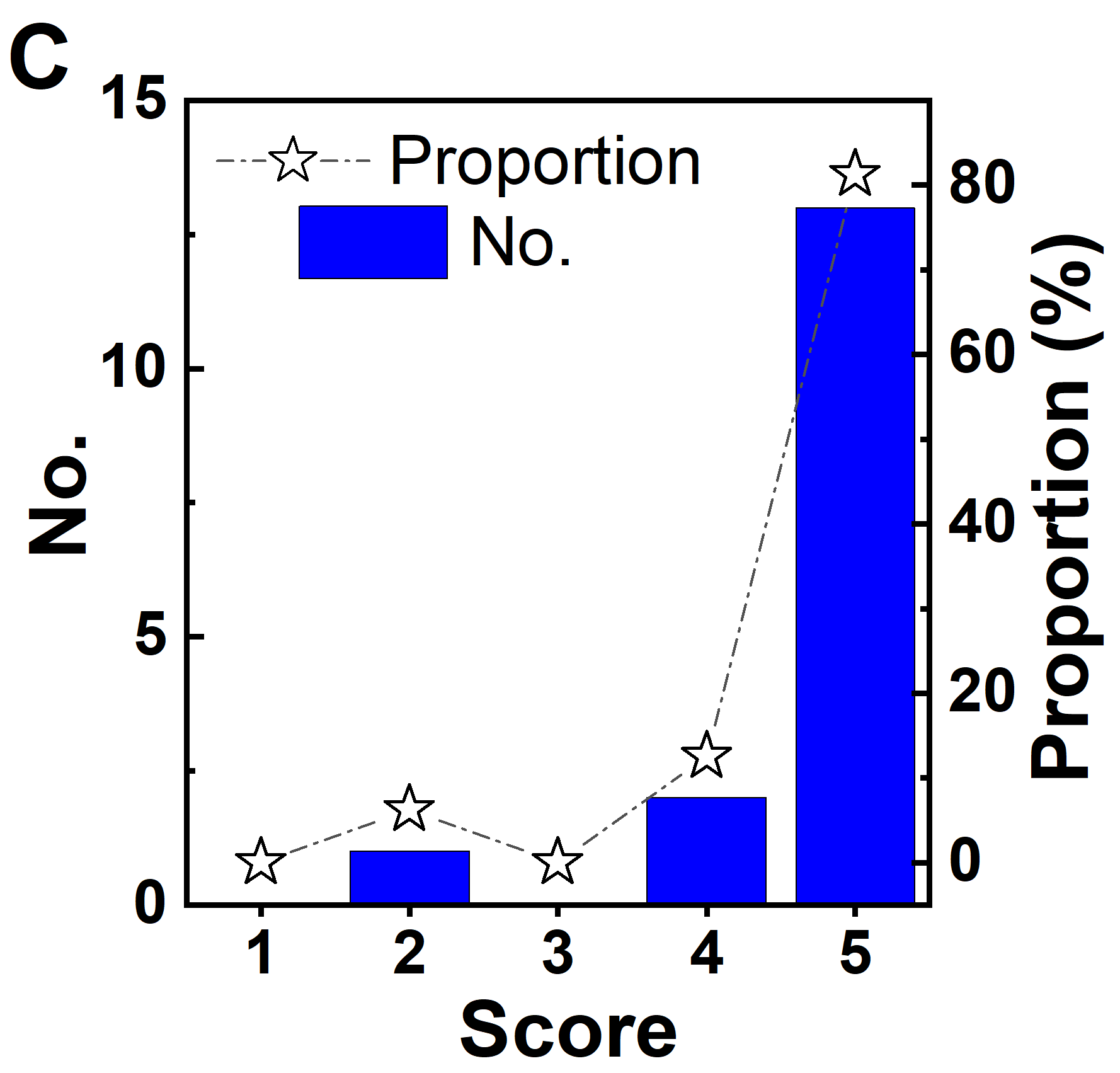

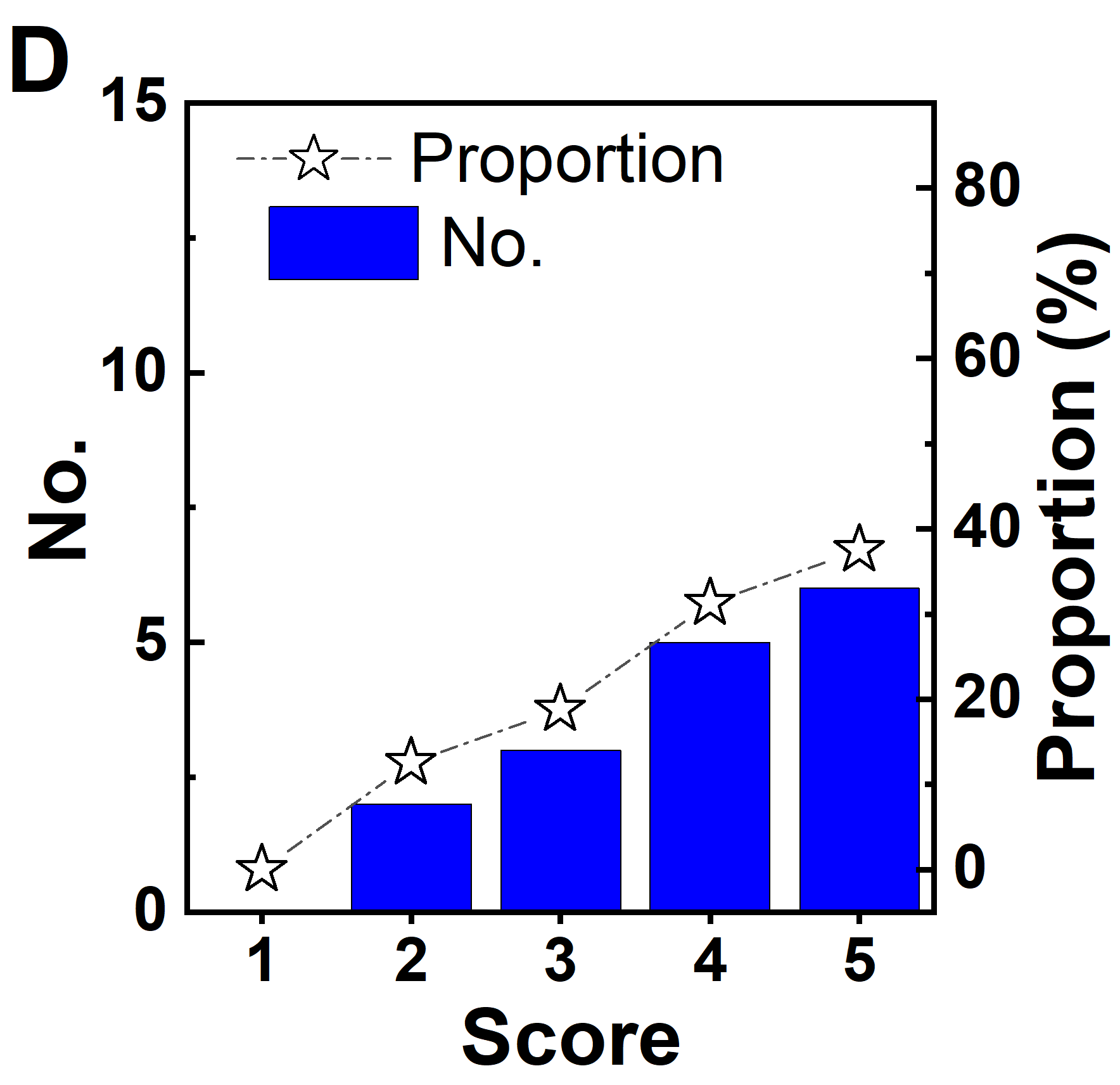


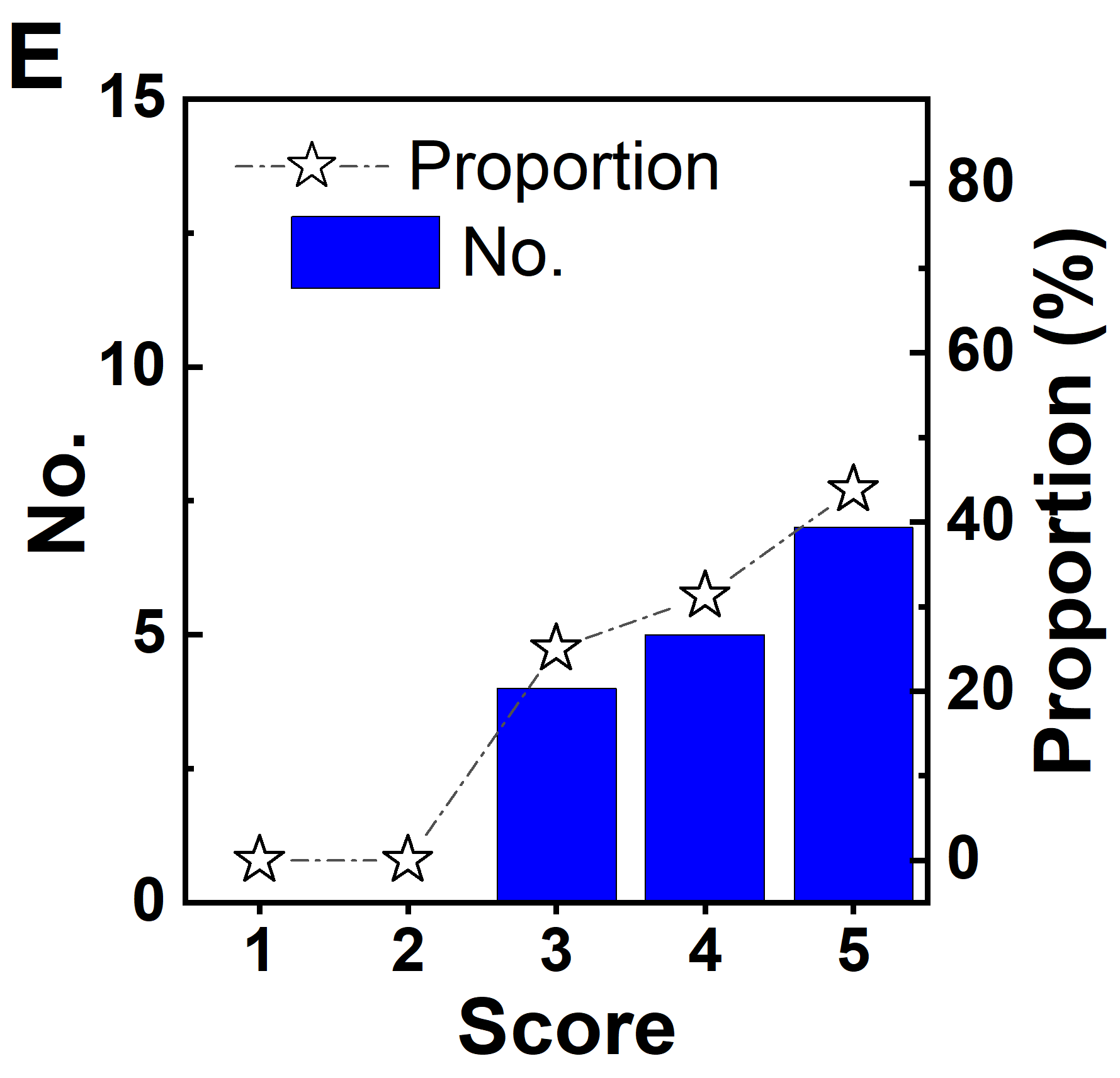


**Fig. S9 ǀ Questionnaire results of comfortability and practicality of the system.** **A**, Smart insoles are comfortable. **B**, The position of laser light is comfortable. **C**, The weight of laser light is acceptable. **D**, The whole set system is easy to wear. And **E**, APP is easy to use. 1-strongly disagree, 2-disagree, 3- nurture, 4-agree, 5-strongly agree.


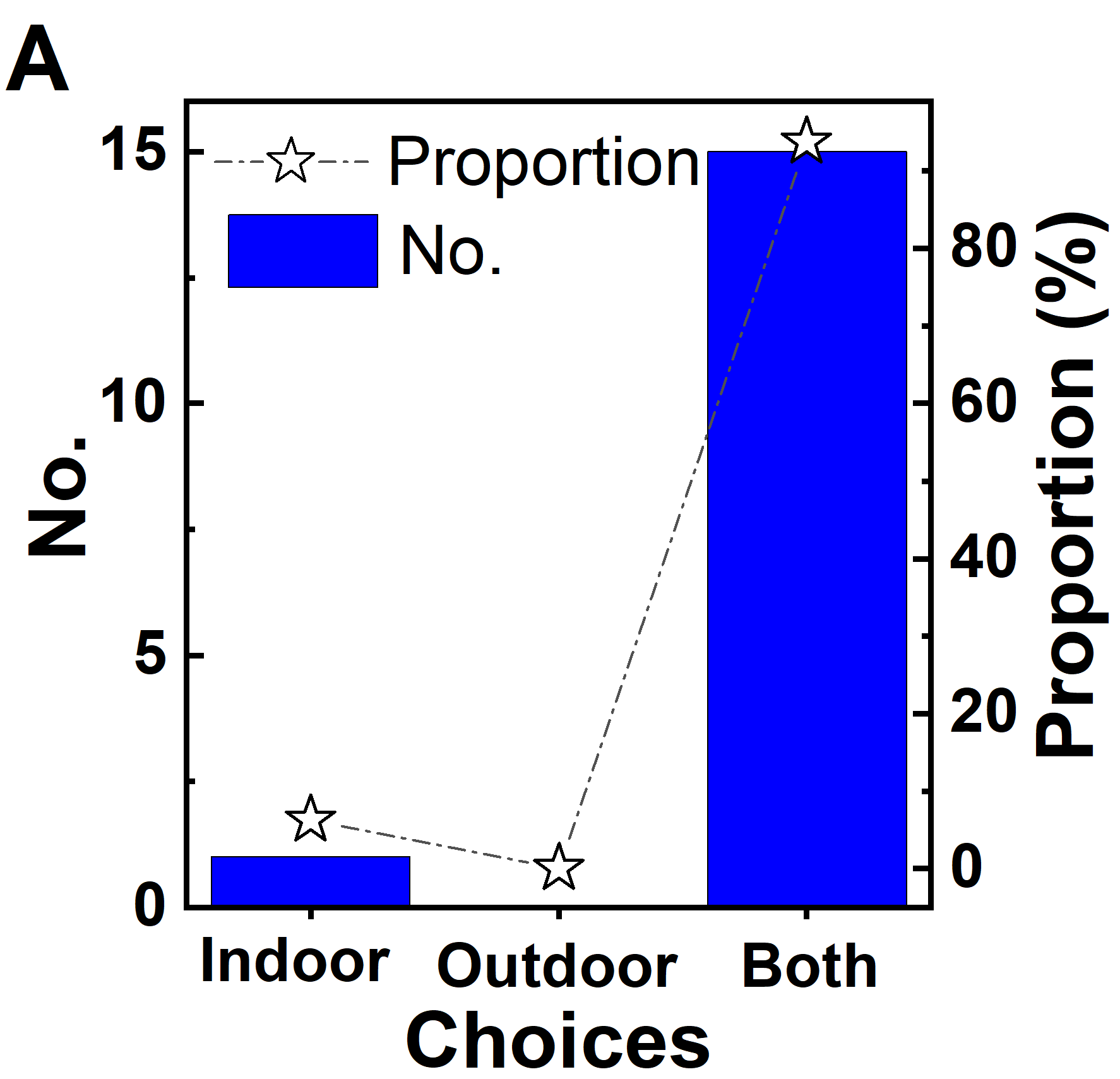

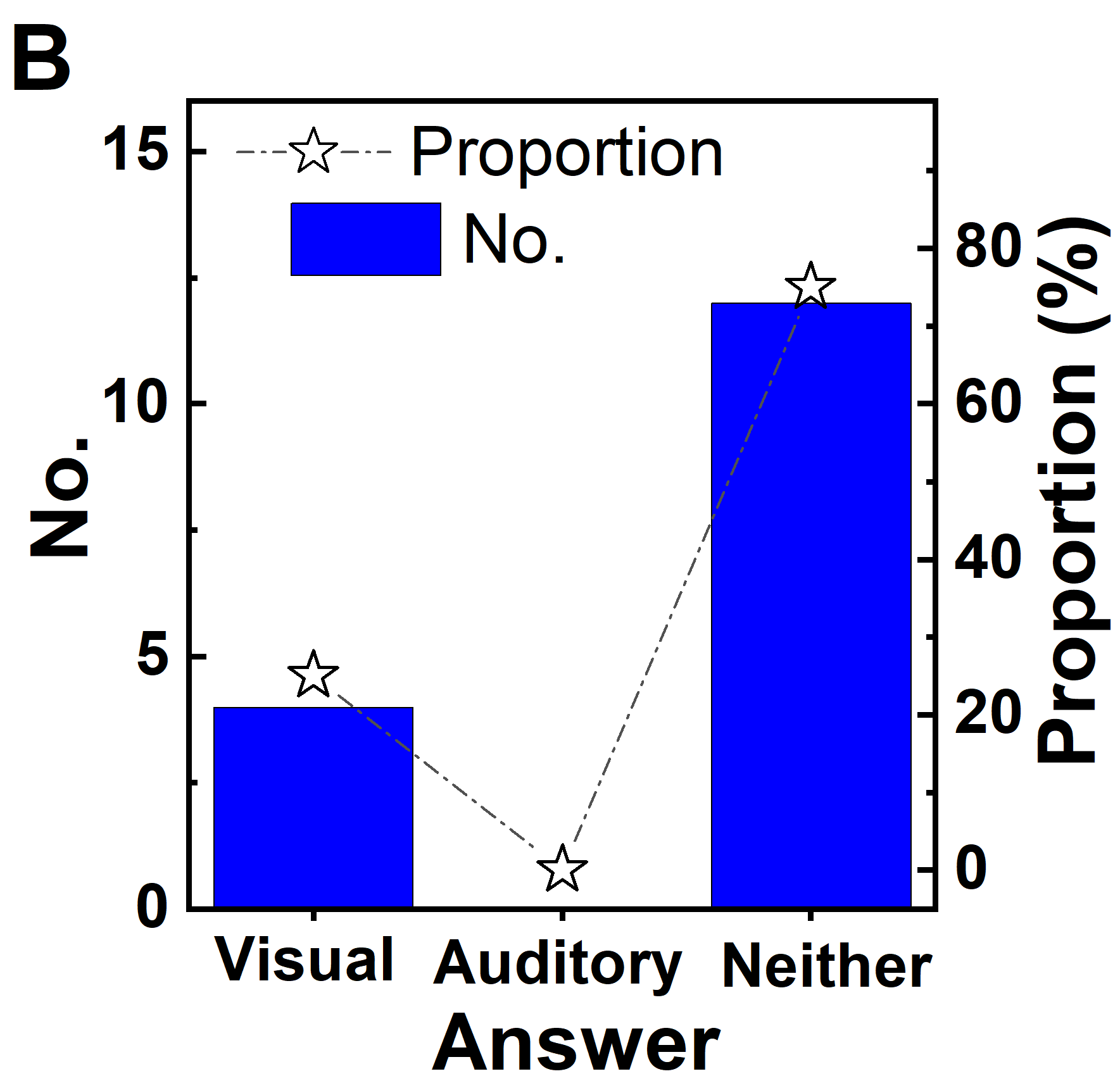


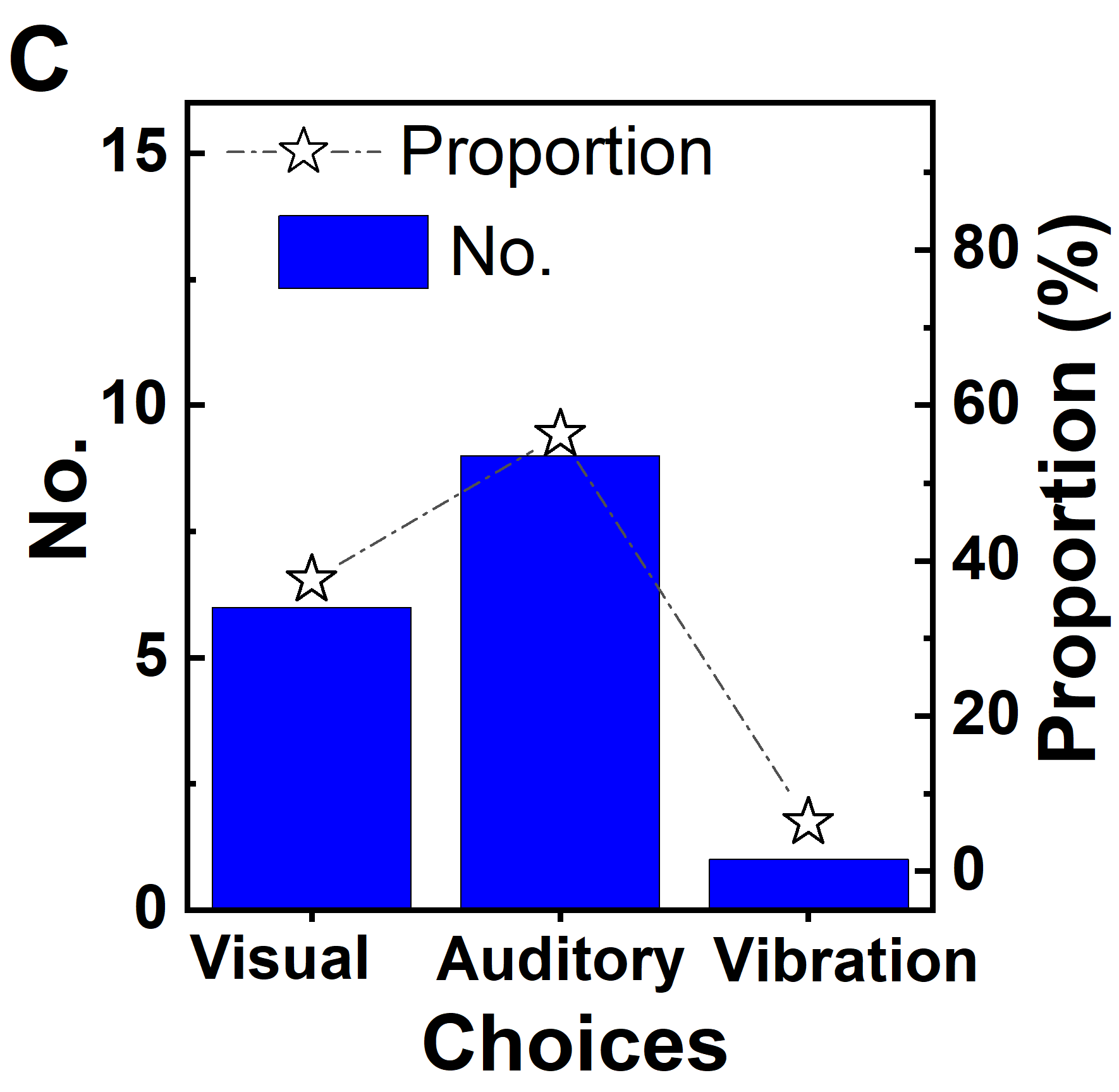

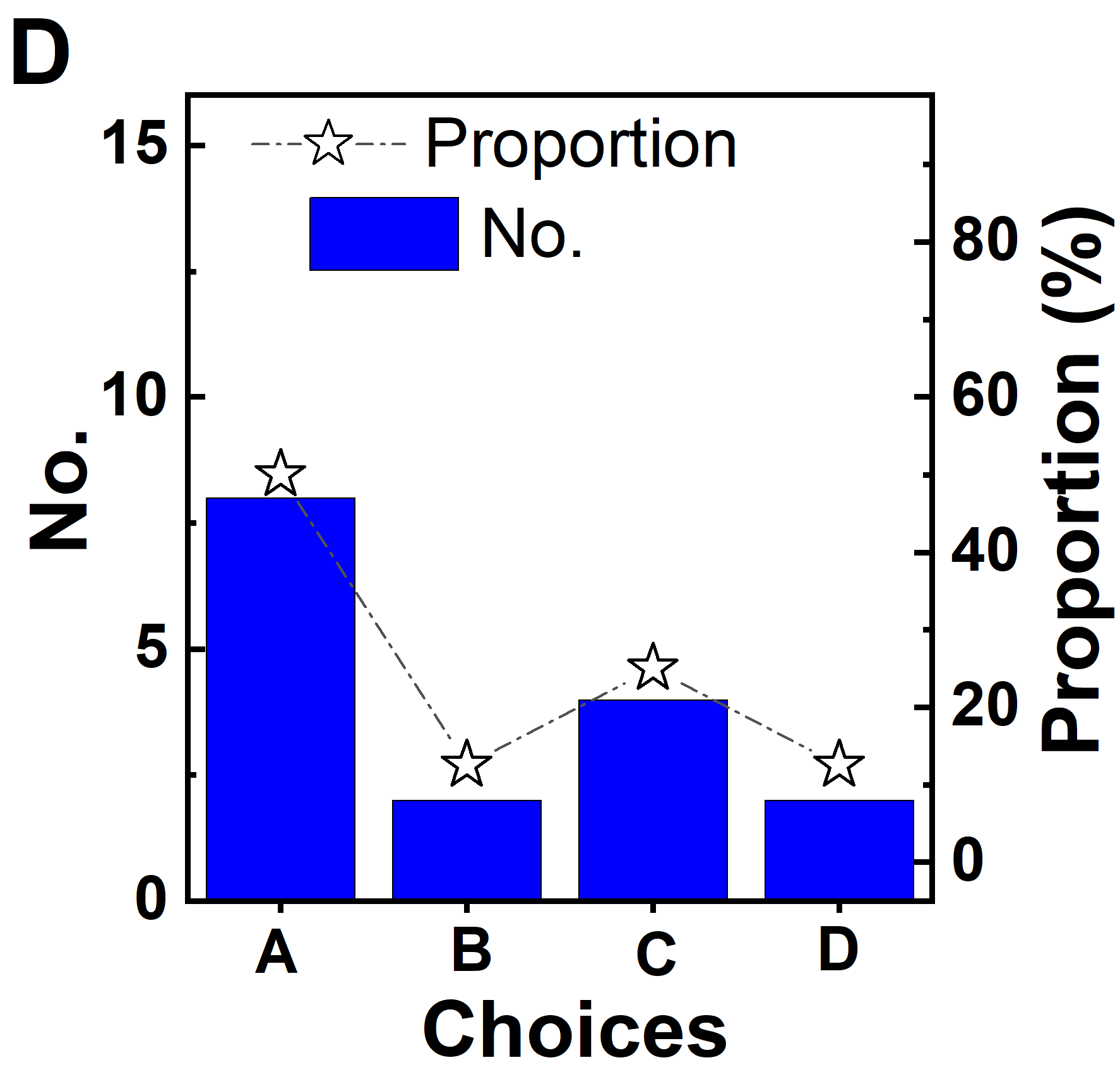


**Fig. S10 ǀ Questionnaire results of suggestion for improvement**. **A**, Environment of the usage. **B**, Did you use other cueing is effective for you? **C**, Which kind of cueing is effective for you? And **D**, Which method do you prefer to cancel the cueing if it is bother you? A-It is acceptable, don’t need to switch off, B-Control by APP manually, C-Switch off by the special movement of a foot, D-Double tap the Bluetooth device like the gestures of earphone.

**Supplementary Tables**

**Table S1** Response time of bare PSUs

| **Initial threshold (kPa)** | | **Test 1 (ms)** | | | | **Test 2 (ms)** | | | | **Test 3 (ms)** | | | | **Average(std) (ms)** |
| --- | --- | --- | --- | --- | --- | --- | --- | --- | --- | --- | --- | --- | --- | --- |
| 20 | Loading | 1 | 1 | 2 | 1 | 1 | 1 | 1 | 1 | 1 | 1 | 1 | 1 | 1(0.3) |
|  | Unloading | 16 | 24 | 24 | 23 | 25 | 23 | 23 | 23 | 23 | 23 | 23 | 23 | 23(2.1) |
| 30 | Loading | 1 | 1 | 1 | 1 | 1 | 1 | 1 | 1 | 1 | 0 | 1 | 1 | 1(0.3) |
|  | Unloading | 25 | 19 | 18 | 17 | 15 | 16 | 16 | 15 | 17 | 16 | 16 | 17 | 17(2.6) |
| 40 | Loading | 1 | 1 | 1 | 1 | 1 | 1 | 1 | 1 | 1 | 1 | 1 | 1 | 1(0.0) |
|  | Unloading | 18 | 19 | 19 | 18 | 19 | 18 | 15 | 18 | 19 | 17 | 18 | 17 | 18(1.1) |

Note: Initial threshold means the design threshold of the bare PSU. Each test has four cycles of compression and release.

**Table S2 Information of subjects**

| **Subject ID** | **Gender** | **Age** | **Weight** | **Height** | **H&Y** | **Shoe size**  **(US)** | **Trial ID** |
| --- | --- | --- | --- | --- | --- | --- | --- |
|  |  | **years** | **kg** | **cm** |  |  |  |
| 1 | Male | 52 | 60 | 169 | 2.5 | 42.0 | 1 |
| 2 | Male | 40 | 55 | 165 | 3.0 | 39.0 | 2,7 |
| 3 | Female | 48 | 55 | 158 | 2.5 | 37.0 | 3 |
| 4 | Female | 61 | 55 | 158 | 2.5 | 37.0 | 4,5 |
| 5 | Male | 76 | 70 | 172 | 2.5 | 42.0 | 6 |
| 6 | Male | 67 | 58 | 165 | 2.5 | 41.0 | 8 |
| 7 | Male | 61 | 74 | 165 | 3.0 | 43.0 | 9 |
| 8 | Male | 61 | 72 | 170 | 3.0 | 41.5 | 10 |
| 9 | Male | 58 | 63 | 168 | 2.5 | 42.0 | 11,14 |
| 10 | Male | 68 | 55 | 165 | 2.0 | 42.0 | 12 |
| 11 | Male | 62 | 71 | 170 | 2.0 | 42.0 | 13 |
| 12 | Male | 61 | 71 | 179 | 2.0 | 42.0 | 15 |
| 13 | Male | 64 | 82 | 177 | 1.5 | 42.5 | 16 |
| 14 | Male | 70 | 48 | 160 | 1.5 | 42.0 | 17 |
| 15 | Male | 66 | 56 | 175 | 2.0 | 42.0 | 18 |
| 16 | Male | 74 | 45 | 155 | 4.0 | 41.0 | 19 |
| 17 | Male | 66 | 67 | 157 | 3.0 | 41.5 | 20,32 |
| 18 | Male | 70 | 62 | 165 | 1.5 | 42.5 | 21 |
| 19 | Male | 62 | 66 | 170 | 1.5 | 42.0 | 22 |
| 20 | Male | 79 | 62 | 170 | 1.5 | 42.0 | 23 |
| 21 | Male | 72 | 58 | 165 | 2.0 | 42.0 | 24 |
| 22 | Male | 60 | 62 | 162 | 3.0 | 41.0 | 27 |
| 23 | Male | 73 | 51 | 155 | 2.0 | 41.0 | 28 |
| 24 | Male | 69 | 74 | 187 | 3.0 | 41.0 | 29,51 |
| 25 | Male | 65 | 60 | 170 | 2.0 | 41.0 | 30 |
| 26 | Male | 58 | 82 | 172 | 1.5 | 41.0 | 31 |
| 27 | Male | 73 | 68 | 158 | 3.0 | 41.0 | 33 |
| 28 | Male | 73 | 53 | 170 | 5.0 | 43.0 | 34,50 |
| 29 | Male | 69 | 65 | 178 | 1.5 | 42.0 | 35 |
| 30 | Female | 65 | 37 | 158 | 3.0 | 38.5 | 36 |
| 31 | Female | 52 | 53 | 158 | 2.0 | 38.0 | 37 |
| 32 | Female | 64 | 42 | 152 | 3.0 | 37.0 | 38 |
| 33 | Female | 61 | 55 | 158 | 2.0 | 38.0 | 39 |
| 34 | Male | 67 | 71 | 165 | 3.0 | 41.0 | 40 |
| 35 | Female | 63 | 45 | 157 | 2.0 | 38.0 | 41 |
| 36 | Female | 68 | 47 | 160 | 1.5 | 37.0 | 42 |
| 37 | Male | 76 | 58 | 160 | 1.5 | 41.5 | 43 |
| 38 | Female | 62 | 57 | 154 | 2.0 | 37.0 | 44 |
| 39 | Male | 72 | 74 | 167 | 2.5 | 42.0 | 45 |
| 40 | Male | 84 | 52 | 158 | 3.0 | 39.0 | 46 |
| 41 | Female | 59 | 52 | 161 | 2.0 | 38.0 | 47 |
| 42 | Female | 66 | 52 | 155 | 1.5 | 40.0 | 48 |
| 43 | Female | 72 | 54 | 157 | 2.0 | 36.0 | 49 |
| 44 | Male | 31 | 60 | 178 | - | 42.0 | 25,26 |

**Table S3** Training and testing results for $\alpha$ and $\beta$

| **Random Subsets** | | **Performance for** $\boldsymbol{\alpha}$ **(%)** | | | **Performance for** $\boldsymbol{\beta}$ **(%)** | | |
| --- | --- | --- | --- | --- | --- | --- | --- |
|  |  | ***Sen*** | ***Sec*** | ***Acc*** | ***Sen*** | ***Sec*** | ***Acc*** |
| 1st | Training | 97.29 | 97.66 | 97.62 | 100.00 | 97.35 | 97.35 |
|  | Testing | 96.82 | 98.19 | 98.05 | 100.00 | 97.16 | 97.16 |
| 2nd | Training | 97.11 | 97.99 | 97.90 | 100.00 | 97.49 | 97.49 |
|  | Testing | 96.97 | 97.91 | 97.81 | 100.00 | 97.04 | 97.04 |
| 3rd | Training | 96.86 | 98.00 | 97.90 | 100.00 | 97.07 | 97.07 |
|  | Testing | 97.18 | 97.90 | 97.82 | 100.00 | 97.40 | 97.40 |
| 4th | Training | 97.29 | 97.66 | 97.62 | 100.00 | 97.35 | 97.35 |
|  | Testing | 96.82 | 98.19 | 98.05 | 100.00 | 97.16 | 97.16 |
| 5th | Training | 96.69 | 97.97 | 97.85 | 100.00 | 97.40 | 97.40 |
|  | Testing | 97.25 | 97.93 | 97.85 | 100.00 | 97.12 | 97.12 |
| Mean ± std | Training | 97.1±0.2 | 97.9±0.3 | 97.8±0.2 | 100.0±0.0 | 97.3±0.2 | 97.3±0.2 |
|  | Testing | 97.0±0.2 | 98.0±0.2 | 97.9±0.2 | 100.0±0.0 | 97.2±0.2 | 97.2±0.2 |

**Table S4** Training and testing results for using $\alpha$ and $\beta$

| **Random Subsets** | | **Performance (%)** | | |
| --- | --- | --- | --- | --- |
|  |  | ***Sen*** | ***Sec*** | ***Acc*** |
| 1st | Training | 97.79 | 94.77 | 95.07 |
|  | Testing | 97.44 | 95.09 | 95.34 |
| 2nd | Training | 97.60 | 95.25 | 95.49 |
|  | Testing | 97.59 | 94.68 | 94.99 |
| 3rd | Training | 97.55 | 94.85 | 95.08 |
|  | Testing | 97.67 | 95.02 | 95.33 |
| 4th | Training | 97.79 | 94.77 | 95.07 |
|  | Testing | 97.44 | 95.09 | 95.34 |
| 5th | Training | 97.02 | 95.14 | 95.31 |
|  | Testing | 97.97 | 94.78 | 95.14 |
| Mean ± std | Training | 97.5 ± 0.5 | 95.0 ±0.3 | 95.2 ±0.3 |
|  | Testing | 97.6 ± 0.4 | 94.9 ±0.3 | 95.2 ±0.3 |

**Table S5** Response time for visual cueing and FoG as long double support

| **No.** | **The *i^th^* trial** | **The *i^th^* test** | **Response time (s)** |
| --- | --- | --- | --- |
| 1 | 39 | 1 | 0.34 |
| 2 | 40 | 3 | 0.44 |
| 3 | 40 | 4 | 0.54 |
| 4 | 40 | 8 | 0.71 |
| 5 | 42 | 2 | 0.24 |
| 6 | 42 | 4 | 0.40 |
| 7 | 42 | 5 | 0.40 |
| 8 | 42 | 7 | 0.20 |
| 9 | 42 | 8 | 0.34 |
| 10 | 46 | 4 | 0.26 |
| 11 | 46 | 4 | 0.36 |
| 12 | 47 | 3 | 0.34 |
| 13 | 47 | 3 | 0.44 |
| 14 | 47 | 4 | 0.36 |
| 15 | 47 | 4 | 0.33 |
| 16 | 47 | 7 | 0.28 |
| 17 | 47 | 7 | 0.43 |
| 18 | 47 | 7 | 0.38 |
| 19 | 49 | 2 | 0.56 |
| 20 | 49 | 3 | 0.23 |
| 21 | 49 | 5 | 0.23 |
| 22 | 49 | 6 | 0.26 |
| Average (std) | - | - | 0.37 (0.12) |
| Max | - | - | 0.71 |
| Min | - | - | 0.23 |

**Note:** There are several tests in one trial. Each continuous walking (two cycles) was a test. And the subject took a rest between the adjacent tests.

**Table S6** Response time for visual cueing and FoG as continuous short swings

| **No.** | **The *i^th^* trial** | **The *i^th^* test** | **Response time (s)** |
| --- | --- | --- | --- |
| 1 | 40 | 1 | 0.37 |
| 2 | 40 | 2 | 0.37 |
| 3 | 40 | 12 | 0.27 |
| 4 | 42 | 8 | 0.30 |
| 5 | 44 | 9 | 0.37 |
| 6 | 45 | 2 | 0.30 |
| 7 | 48 | 5 | 0.73 |
| 8 | 48 | 6 | 0.27 |
| 9 | 50 | 5 | 0.27 |
| 10 | 51 | 3 | 0.47 |
| 11 | 51 | 5 | 0.37 |
| Average (std) | - | - | 0.37 (0.14) |
| Max | - | - | 0.73 |
| Min |  |  | 0.27 |

**Note:** There are several tests in one trial. Each continuous walking (two cycles) was a test. And the subject took a rest between the adjacent tests.

**Reference**

Lorenzi, P., Rao, R., Romano, G., Kita, A., & Irrera, F. (2016). Mobile devices for the real-time detection of specific human motion disorders. *IEEE Sensors Journal*, *16*(23), 8220-8227. <https://doi.org/10.1109/JSEN.2016.2530944>

Mikos, V., Heng, C.-H., Tay, A., Yen, S.-C., Chia, N. S. Y., Koh, K. M. L., Tan, D. M. L., & Au, W. L. (2019). A Wearable, Patient-Adaptive Freezing of Gait Detection System for Biofeedback Cueing in Parkinson's Disease. *IEEE transactions on biomedical circuits and systems*, *13*(3), 503-515. <https://doi.org/10.1109/TBCAS.2019.2914253>
